# A Novel Nanozyme to Enhance Radiotherapy Effects by Lactic Acid Scavenging, ROS Generation, and Hypoxia Mitigation

**DOI:** 10.1002/advs.202403107

**Published:** 2024-05-05

**Authors:** Yiran Yao, Ru Xu, Weihuan Shao, Ji Tan, Shaoyun Wang, Shuhan Chen, Ai Zhuang, Xuanyong Liu, Renbing Jia

**Affiliations:** ^1^ Department of Ophthalmology, Ninth People's Hospital Shanghai JiaoTong University School of Medicine Shanghai 200011 P. R. China; ^2^ Shanghai Key Laboratory of Orbital Diseases and Ocular Oncology Shanghai 200011 P. R. China; ^3^ State Key Laboratory of High Performance Ceramics and Superfine Microstructure Shanghai Institute of Ceramics Chinese Academy of Sciences Shanghai 200050 P. R. China

**Keywords:** lactic acid scavenging, layered double oxides, nanozyme, radiotherapy sensitization, uveal melanoma

## Abstract

Uveal melanoma (UM) is a leading intraocular malignancy with a high 5‐year mortality rate, and radiotherapy is the primary approach for UM treatment. However, the elevated lactic acid, deficiency in ROS, and hypoxic tumor microenvironment have severely reduced the radiotherapy outcomes. Hence, this study devised a novel CoMnFe‐layered double oxides (LDO) nanosheet with multienzyme activities for UM radiotherapy enhancement. On one hand, LDO nanozyme can catalyze hydrogen peroxide (H_2_O_2_) in the tumor microenvironment into oxygen and reactive oxygen species (ROS), significantly boosting ROS production during radiotherapy. Simultaneously, LDO efficiently scavenged lactic acid, thereby impeding the DNA and protein repair in tumor cells to synergistically enhance the effect of radiotherapy. Moreover, density functional theory (DFT) calculations decoded the transformation pathway from lactic to pyruvic acid, elucidating a previously unexplored facet of nanozyme activity. The introduction of this innovative nanomaterial paves the way for a novel, targeted, and highly effective therapeutic approach, offering new avenues for the management of UM and other cancer types.

## Graphical abstract

Tackling the problem of intrinsic radiation resistance in Uveal Melanoma (UM), this study tailored a CoMnFe‐layered double oxides (LDO) nanosheet as a versatile nanozyme. By synergistically targeting lactic acid clearance, reactive oxygen species amplification, and oxygen production, CoMnFe‐LDO distinctly enhances the radiotherapy effect of UM in vitro and in vivo. This study presents a novel strategy of designing LDO nanosheets with multienzyme activities for radiosensitization, which has good potential in cancer therapy.

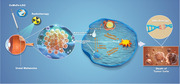

## 1 Introduction

Uveal melanoma (UM) is the most common primary intraocular malignant tumor in adults, with a grim prognosis and 5‐year mortality rate ranging from 37 to 50%.^[^ ^1^ ^]^ Radiotherapy is the primary and preferred approach for UM;^[^ ^2^ ^]^ however, it is often associated with treatment failure or recurrence. These challenges are rooted in the intrinsic radiation‐resistant nature of UM. The high radiation resistance of UM translates into a narrow therapeutic window, often necessitating radiation doses of 60–100 Gy. These high doses result in a series of radiation‐related complications, such as neovascular glaucoma, radiation retinopathy, radiation optic neuropathy, vision impairment, and, in extreme cases, a second enucleation due to complications.^[^ ^3^ ^]^ Therefore, addressing the clinical difficulties posed by high radiation resistance through radiotherapy sensitization is important for improving patient outcomes and minimizing side effects.

Ionizing radiation in tumor radiotherapy triggers cell death by directly damaging biomolecules, such as DNA, and indirectly creating reactive oxygen species (ROS).^[^ ^3^, ^4^ ^]^ Consequently, augmenting ROS production and effectiveness during radiotherapy remains the preferred approach for radiation enhancement. Hypoxia contributes significantly to resistance to treatment, including radiotherapy.^[^ ^5^ ^]^ On the one hand, the hypoxic property of the tumor microenvironment (TME) is not conducive to ROS generation during radiotherapy.^[^ ^6^ ^]^ In contrast, oxygen can interact with ROS through the “oxygen effect” to produce more stable and irreparable damage to biomolecules.^[^ ^6^ ^]^ Unfortunately, the “radiation damage fixation” effect of oxygen within the hypoxic TME is ineffective. Alleviating TME hypoxia has been employed as an efficient strategy for radiation sensitization.^[^ ^7^ ^]^ Existing literature primarily identifies several key mechanisms by which nanoparticles enhance the efficacy of radiotherapy: alleviating hypoxia in the TME, promoting the generation of ROS, reducing glutathione levels, and leveraging the properties of heavy metal ions.^[^ ^8^ ^]^ However, these approaches primarily focus on adjusting the physical and chemical properties of the materials and often overlook the unique characteristics inherent to the tumors themselves. Radiosensitizing strategies should comprehensively address other emerging and complex mechanisms associated with radiotherapy resistance. However, up to now, few radiosensitizing strategies have successfully integrated multiple promising targets to achieve synergistic and cascading effects.

Recent seminal studies have highlighted the significance of integrated metabolic reprogramming as a central mechanism underlying radiation resistance.^[^ ^9^ ^]^ Metabolic alterations in the TME counteract and diminish the radiotherapy cytotoxicity. Within metabolic integration, lactic acid in the TME has been identified as a key player in both aerobic glycolysis and the tricarboxylic acid cycle.^[^ ^10^ ^]^ Historically regarded as a byproduct of cellular metabolism and dubbed the “ugly duckling of energy metabolism,”^[^ ^10^, ^11^ ^]^ lactic acid has recently gained recognition for its role in cancer biology. Emerging research has elucidated its multifaceted functions in the TME, including immune evasion, angiogenesis, and treatment resistance,^[^ ^12^ ^]^ Elevated levels of lactic acid have been implicated in the promotion of radiotherapy resistance through diverse mechanisms in numerous tumor types, such as rectal, pancreatic, and lung cancers.^[^ ^9^, ^13^ ^]^ It has the potential to serve as a biomarker for predicting unfavorable radiotherapy outcomes.^[^ ^9^, ^13^, ^14^ ^]^ Lactic acid primarily generates reductive metabolites that effectively neutralize ROS such as superoxide and hydroxyl radicals, which are key mediators of radiotherapy.^[^ ^9^, ^13^ ^]^ Furthermore, the impact of lactic acid on DNA damage repair‐related genes through histone acetylation and lactic acid dehydrogenase A, as well as its role in promoting the active repair of radiotherapy damage, compromises the effectiveness of radiotherapy.^[^ ^15^ ^]^ Therefore, lactic acid scavenging is vital to enhance the effect of radiotherapy.

Contemporary strategies for lactic acid scavenging predominantly rely on three approaches: natural enzymes that metabolize lactic acid, small‐molecule inhibitors that target these enzymes, and inhibitors that block lactic acid transport. Despite their potential, small‐molecule inhibitors have off‐target effects.^[^ ^16^ ^]^ While enzymes specializing in lactic acid metabolism are highly efficient, their application can be limited by lower stability in harsh environments, such as acidic conditions often encountered within the tumor microenvironment.^[^ ^17^ ^]^ As promising alternatives, catalytically active nanomaterials, commonly referred to as “nanozymes,” have garnered attention.^[^ ^18^ ^]^ They offer several advantages including enhanced catalytic efficiency, resilience to harsh conditions, long‐lasting efficacy, and targeted delivery. These advantages endow nanozymes with multifaceted and robust therapeutic capabilities.^[^ ^18^ ^]^ However, the challenges of utilizing nanozymes to oxidize lactic acid to pyruvic acid within the human body are substantial, primarily because of high‐energy carbon–hydrogen bonds, thereby limiting the progress in nanozyme development for lactic acid clearance.^[^ ^19^ ^]^ An ideal nanozyme for lactate clearance should possess high specific surface areas and robust electron transfer capabilities.^[^ ^20^ ^]^ The layered double oxide (LDO) harnesses the catalytic benefits of transition metals facilitating catalytic reactions.^[^ ^21^ ^]^ The 2D structure of LDO maximizes the surface area, thus increasing the number of active sites available for catalysis.^[^ ^21^ ^]^ Importantly, LDO has strong electron transfer capability, and has shown promising enzyme‐mimetic activities, such as peroxidase‐mimetic and oxidase‐mimetic activities.^[^ ^22^ ^]^ These characterizations endow LDO with the ability to scavenge lactic acid from lactate‐rich TMEs and maintain their activity over an extended period.

In this study, we engineered a multifaceted nanozyme CoMnFe‐layered double oxide (LDO) with a lactate‐clearing capability to address the resistance encountered in UM. LDO exhibits TME responsiveness by catalyzing hydrogen peroxide and generating radiosensitizing agents. Leveraging its transition‐metal constituents, LDO catalyzes the conversion of hydrogen peroxide into more toxic hydroxyl radicals, thereby increasing the efficacy of radiotherapy. Furthermore, LDO, as a manganese‐based material, catalyzes the decomposition of hydrogen peroxide to oxygen and mitigates the hypoxic TME. The oxygen generated through LDO catalysis plays a dual role, aiding lactic acid clearance and further mitigating hypoxic conditions, thus forming a self‐sustaining cycle. This work focused on the key issues in UM radiotherapy and considered the comprehensive metabolic mechanisms of radiotherapy resistance. This study offers a highly active, TME‐responsive, and long‐residence nanomaterial, providing a novel cascading approach for enhancing radiotherapy sensitization in UM and other cancer types that potentially benefit from radiotherapy enhancement.

## 2 Results and Discussion

### 2.1 Preparation and Characterization of LDO

**Figure 1:**
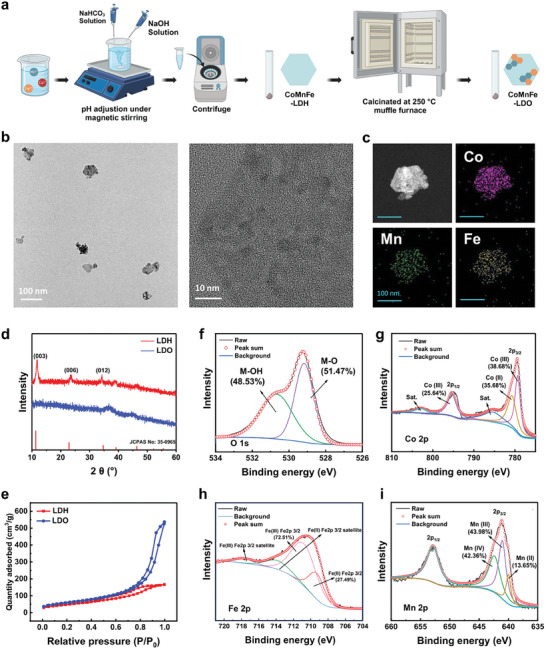
Characterization and physicochemical properties of LDO nanosheets. a) Visual diagram illustrating LDO nanosheet preparation process. b) TEM images and c) TEM elemental distribution mapping. d) and e) Surface area analysis using the Brunauer–Emmett–Teller method. High‐resolution X‐ray photoelectron spectroscopy (XPS) spectrum of O f), Co g), Fe h), and Mn i).

The synthesis culminated in LDO, following the calcination of CoMnFe‐layered double hydroxides (LDH) at 250 °C, and was designated as LDO (**Figure** **1**a). Transmission Electron Microscope (TEM) and element mapping images (Figure 1b,c) revealed an incomplete regular hexagonal structure of LDO, and the Co, Mn, and Fe elements were uniformly distributed. Well‐crystallized LDH, the precursor to LDO, typically exhibits a regular hexagonal shape. However, during the calcination process that transforms LDH to LDO, the decomposition of carbonate ions occurs, leading to the production of carbon dioxide gas. The extensive release of carbon dioxide gas disrupts the crystal structure, resulting in a deterioration of crystallinity. Consequently, this process leads to the irregular morphology and size variations observed in the TEM images of LDO. Through Energy‐Dispersive X‐ray Spectroscopy (EDS) analysis, we have determined the elemental atomic ratios in LDO to be: Mn at 6.04%, Fe at 2.51%, and Co at 18.66%. The XRD pattern of LDH is characterized by distinct peaks at 003, 006, 012, etc., which are in agreement with the standard peaks of LDH as per the Powder Diffraction File (PDF), reflecting the successful preparation of the LDH (Figure 1d). The characteristic peaks of LDO disappeared after calcine treatment of LDH (Figure 1d). Brunauer–Emmett–Teller analysis showed that the specific surface area increased from 146.20 m^2^ g^−1^ for LDH to 212.72 m^2^ g^−1^ for LDO (Figure 1e). This enlarged surface area may be the cornerstone for the enhanced catalytic activity of the nanosheets. X‐ray photoelectron spectroscopy (XPS) confirmed the presence of Co, Mn, Fe, O, and C on the near surfaces, and high‐resolution spectra elucidated the chemical states of Co, Mn, and Fe within the LDO (Figure 1f–i). The identification of Co^2+^/Co^3+^, Fe^2+^/Fe^3+^, and Mn^2+^/Mn^4+^ redox couples within the LDO nanosheets, which may contribute to catalyze the H_2_O_2_ to ROS and O_2_ generation, and consume the lactic acid.

### 2.2 The Multifaceted Functions of LDO Nanosheets

**Figure 2:**
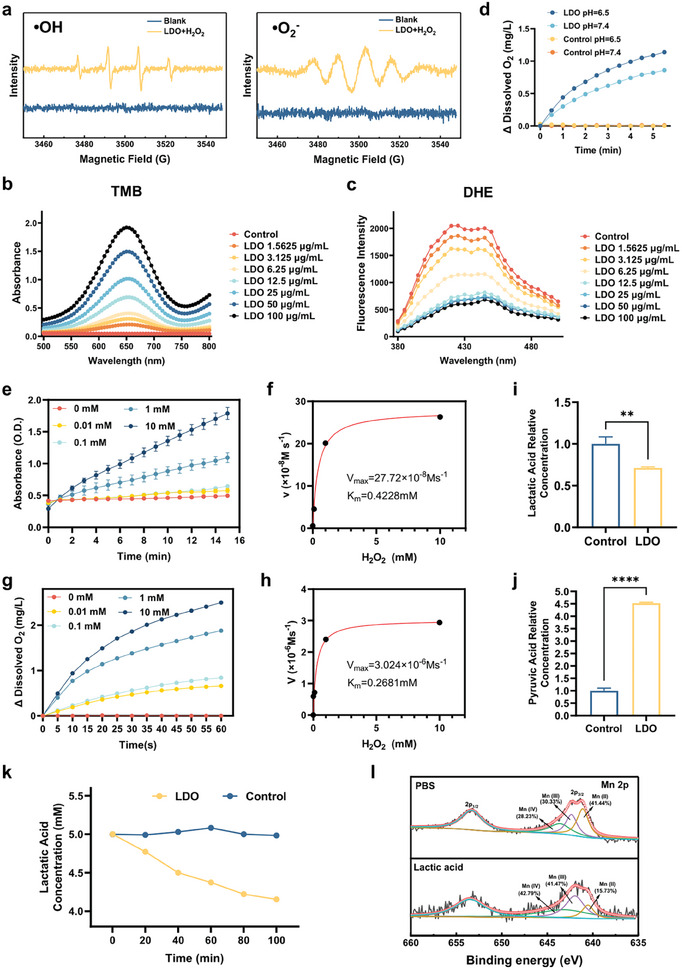
Multifaceted functions of LDO nanosheets: lactic acid scavenging, ROS amplification, and oxygen production. a) Electron spin resonance (ESR) spectra. b) TMB oxidation spectral assay. c) DHE fluorescence spectroscopy assay. d) Quantification of oxygen generation. e) Peroxidase‐like activity kinetics using TMB oxidation assay. f) Michaelis‐Menten kinetics curve of Peroxidase‐like activity. g) Catalase‐like activity kinetics using TMB oxidation assay. h) Michaelis–Menten kinetics curve of catalase‐like activity. i) Lactic acid concentration after co‐incubation with LDO nanosheets. Data represent three replicates. j) Pyruvic acid concentration after co‐incubation with LDO nanosheets. Data represent three replicates. k) The lactic acid consumption curve. l) High‐resolution XPS spectra depicting the Mn 2p orbitals. Abbreviations: PBS: phosphate‐buffered saline.

These capabilities including clearance of lactic acid, amplification of reactive ROS, and generation of oxygen of LDO were evaluated. Electron spin resonance (ESR) assays show that LDO facilitated the conversion of hydrogen peroxide (H_2_O_2_) to •OH and superoxide radicals (•O_2_^−^) (**Figure** **2**a). The 3,3′,5,5′‐tetramethylbenzidine (TMB) can be oxidized by hydroxyl radicals to form oxidized TMB (oxTMB), which exhibits a characteristic absorption peak at 652 nm. We also assessed the capacity of LDO to generate hydroxyl radicals under both the neutral and acidic conditions typically found in the TME using a TMB oxidation assay, hydroxyl radical (•OH) production was observed (Figure S1, Supporting Information). With increasing LDO concentration, there is a corresponding increase in the absorption at 652 nm, indicating that the ability of LDO to catalyze the conversion of hydrogen peroxide into hydroxyl radicals increases with LDO concentration (Figure 2b). Dihydroethidium (DHE) is a commonly used superoxide anion fluorescence detection probe. As more superoxide anions are generated, the characteristic twin‐peak fluorescence spectrum becomes more pronounced. Our results show that as the concentration of LDO increases from 0 to 12.5 µg mL^−1^, its ability to catalyze hydrogen peroxide into superoxide anions continuously increases. Beyond 12.5 to 100 µg mL^−1^, there was no significant change in catalytic ability (Figure 2c). The efficient ROS generation may be attributed to the homogeneously dispersed oxides on the LDO surface, its expansive specific surface area, and the augmented concentration of Mn^2+^. Experiments utilizing both TMB Oxidation Assay (Figure S2a, Supporting Information) and DHE Fluorescence Assay (Figure S2b, Supporting Information), show that LDO under radiation conditions produces more hydroxyl radicals and superoxide anions compared to non‐radiation conditions. This suggests that LDO has significant potential as a radiosensitizer.

Simultaneously, LDO converts H_2_O_2_ into oxygen (O_2_), potentially mitigating the low‐oxygen conditions often present in the TME. Accordingly, we assessed the generation of O_2_ at pH 6.5 in the presence of 1 mm H_2_O_2_ using a dissolved oxygen meter (Figure 2d). The results indicated that LDO is particularly effective in acidic environments, underscoring its adaptability to the varying conditions of the TME.

The LDO demonstrates both catalase‐ and peroxidase‐like activities. Therefore, the enzymatic behaviors of LDO have been examined, focusing on their catalase‐ and peroxidase‐like activities. For the peroxidase‐like activity kinetics, we utilized the TMB oxidation assay to investigate the kinetic properties of LDO. Our results align well with the Michaelis‐Menten kinetics (Figure e‐f). The calculated maximum initial velocity (*V*_max_) and Michaelis constant (K_m_) were 27.72 × 10^−8^ Ms^−1^ and 0.4228 mM, respectively. Furthermore, the relationship between enzyme activity and various parameters has been examined (Figure S3, Supporting Information).

Our experiments also revealed that LDO exhibits significant catalase‐like activity, converting hydrogen peroxide prevalent in the tumor microenvironment into water and oxygen, thereby alleviating hypoxia. We used a dissolved oxygen meter to measure oxygen generation, characterizing the kinetics of LDO (50 µg mL^−1^) catalase‐like activity (Figure 2g,h). The data fit well to Michaelis‐Menten kinetics, with a maximum initial velocity (*V*_max_) of 3.024 × 10^−6^ Ms^−1^ and a Michaelis constant (K_m_) of 0.2681 mM. Furthermore, the relationship between enzyme activity and various parameters has been examined (Figure S4, Supporting Information).

As shown in Figure 2i,j, we employed gas chromatography‐mass spectrometry (GC‐MS) qualitative analysis to verify the changes in lactate and pyruvate concentrations driven by LDO. Our findings indicated that after a 12 h reaction with 50 µg mL^−1^ LDO in a solution containing 3 mm lactate, the lactate concentration decreased to 71.23% of its original level (Figure 2i). Concurrently, the pyruvate concentration increased to 4.52 times its initial amount (Figure 2j). These results indicate the potential of LDO to metabolize lactic acid into pyruvic acid.

Lactic acid depletion kinetics experiments have also been conducted using a concentration of 50 µg mL^−1^ LDO (Figure 2k). Our results indicate that ≈16.67% of lactic acid was depleted within the first 100 min of the reaction.

The high‐resolution spectra of Co, Mn, Fe, and O of the LDO immersed in phosphate‐buffered saline (PBS) and lactic acid solution are shown (Figure 2l; Figure S5, Supporting Information). Although the valence states of Co and Fe remained unaltered after the incubation, the valence state of Mn shifted from Mn^2+^ to Mn^4+^. Concurrently, the content of metal‐hydroxyl (M‐OH) groups increased, indicating the active participation of Mn in catalyzing lactic acid transformation. This increase in M‐OH content could be attributed to the “memory effect” of LDO.

The versatile nanozyme of targeting lactic acid scavenging, ROS amplification, and oxygen production endows LDO with the potential of radiotherapy enhancement.

### 2.3 Mechanistic Insight into Lactic Acid Metabolism by LDO Nanosheets

**Figure 3:**
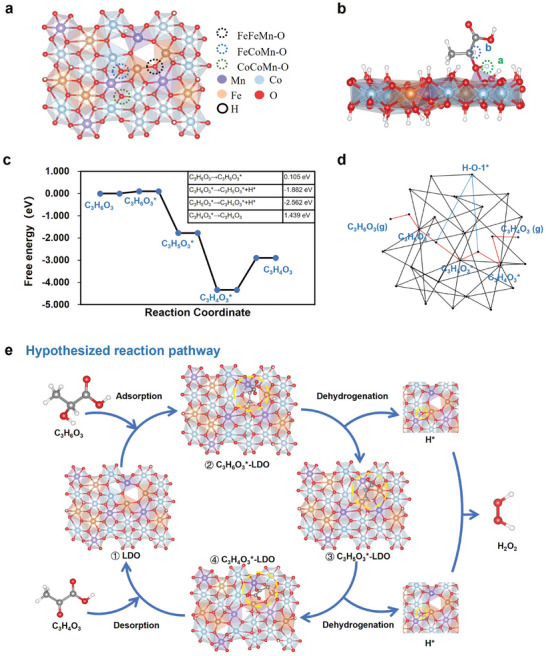
Mechanistic insights into lactic acid metabolism using LDO nanosheets. a) Schematic of the LDO model used for density functional theory (DFT) calculations. b) Labeling of the hydrogen atom bonded to the hydroxyl group on the beta carbon as “a‐H”, and the hydrogen atom on the beta carbon itself as “b‐H”. c) Overview of the reaction processes and the corresponding free energy involved in the catalytic oxidation of lactate to pyruvate. d) Computational exploration of the optimal reaction trajectory. e) Proposed mechanism for lactic acid scavenging by the LDO nanosheets.

To elucidate the potential catalytic mechanism of LDOs in the conversion of lactic acid to pyruvic acid, we constructed an LDO model (**Figure** **3**a). As the model shows, every oxygen atom in the LDO is coordinated to three metal atoms. For clarity, we have designated the oxygen atom bonded to two Fe atoms and one Mn atom as FeFeMn─O. Similarly, the oxygen atoms bonded to other combinations of metal atoms were labeled FeCoMn─O and CoCoMn─O (Figure 3a). To further clarify the chemical structure and eliminate ambiguities, we labeled the hydrogen atom on the hydroxyl group attached to the beta carbon as “a‐H” and the hydrogen atom on the beta carbon as “b‐H” (Figure 3b).

TEM revealed structural defects in the LDO. Therefore, various oxygen vacancy formation scenarios based on this simulation model were considered (Figure S6, Supporting Information). The simulation model with the FeFeMn─O vacancy scenario was selected for further computational calculations because it had the lowest vacancy formation energy (Figure 3a; Figure S6, Supporting Information). Density of states (DOS) analysis was conducted to illustrate the asymmetric DOS feature, which indicates the catalytic potential of LDO (Figure S7, Supporting Information).

Based on first‐principles density functional theory (DFT) calculations, we explored the lactic‐to‐pyruvic acid transformation reaction pathway within this specific LDO simulation model. Multiple variables were considered: possible adsorption sites; different sequences of lactate dehydrogenation (“a‐H” or “b‐H”); post‐dehydrogenation hydrogen atom adsorption locations (Figure S8, Supporting Information); and different intermediates formed. From a set of 2587 plausible catalytic pathways, an energetically favorable route was identified (Figure 3c,d). Thus, we propose the following catalytic pathway for LDO, mimicking the Ping‐Pong mechanism, based on the thermodynamic Mn atom formation of natural LOX (Figure 3e).^[^ ^18^ ^]^ In this optimal path, lactic acid was initially adsorbed onto the FeFeMn─O vacancy, transitioning to an adsorbed state. The hydrogen atom labeled a‐H is first dehydrogenated, resulting in an intermediate compound (C_3_H_5_O_3_*). Subsequently, the b‐H atom was removed, forming another intermediate (C_3_H_4_O_3_*). Eventually, the C_3_H_4_O_3_* intermediate was desorbed from the simulation model, completing the transformation of lactic acid to pyruvic acid. The reaction process is as follows:

(1) $$C_{3}H_{6}O_{3}\to C_{3}H_{6}O_{3}*\;\left(0.105\;\mathrm{eV}\right)$$

(2) $$C_{3}H_{6}O_{3}*\to C_{3}H_{5}O_{3}*+H*\;\left(-1.882\;\mathrm{eV}\right)$$

(3) $$C_{3}H_{5}O_{3}*\to C_{3}H_{4}O_{3}*+H*\;\left(-2.562\;\mathrm{eV}\right)$$

(4) $$C_{3}H_{4}O_{3}*\to C_{3}H_{4}O_{3}\left(1.439\;\mathrm{eV}\right)$$

A charge density difference analysis was performed to evaluate the charge transfer, which further confirmed the potential of a successful catalytic process (Figure S9, Supporting Information).

### 2.4 The Biocompatibility and Bioactivity of the LDO Nanosheets

**Figure 4:**
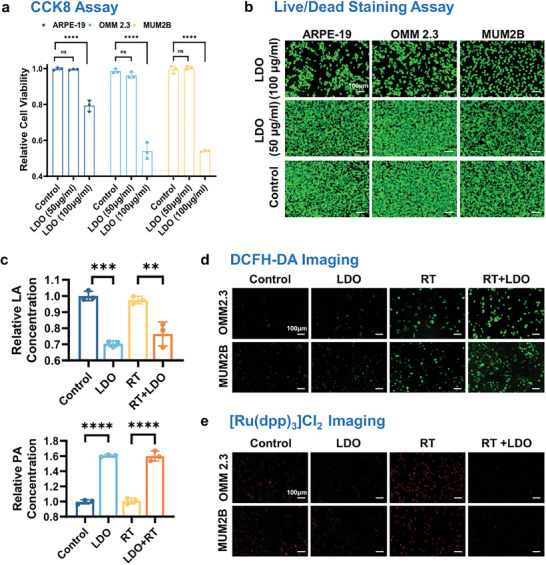
Testing biocompatibility and bioactivity of LDO nanosheets. a) CCK8 assay results showing the proliferative activity of normal ARPE19 cells and tumor cells (OMM2.3, MUM2B) in the presence or absence of LDO nanosheets, with untreated cells serving as controls. Data represent three biological replicates. b) Viability of cells treated with LDO nanosheets versus untreated controls as indicated by live/dead staining. Green fluorescence indicates live cells. Representative images of three experimental replicates are shown. Scale bar: 100 µm. c) Relative lactic acid and pyruvic acid concentrations in OMM2.3 cells under various treatments. d) Intracellular ROS levels in cells exposed to LDO nanosheets as determined by dichlorodihydrofluorescein diacetate (DCFH‐DA) fluorescence imaging. Representative images of three experimental replicates are shown. Scale bar: 100 µm. e) [Ru(dpp)_3_]Cl_2_ fluorescence imaging of cells exposed to LDO nanosheets compared with untreated controls, shedding light on their impact on intracellular oxygen levels. Representative images of three experimental replicates are shown. Scale bar: 100 µm. Abbreviations: LA: lactic acid; RT: radiotherapy.

To evaluate the safety profile of the LDO nanosheets, we employed a CCK8 assay to investigate their effects on cell viability. Normal retinal pigment epithelial cells (ARPE19) and UM cell lines (OMM2.3, MUM2B) were exposed to varying concentrations of LDO nanosheets (0, 50 µg mL^−1^, and 100 µg mL^−1^). Our data revealed that cell viability remained largely unaffected at 50 µg mL^−1^ after 72 h of exposure, confirming minimal cytotoxicity (**Figure** **4**a). The live/dead staining assay demonstrated consistent results with 50 µg mL^−1^ LDO nanosheets showing favorable biocompatibility (Figure 4b). In addition to high cell viability, the overall morphology of live cells remained typical and showed no visible signs of distress or damage, reinforcing the low cytotoxicity of LDOs. These findings suggest that LDO nanosheets display excellent biocompatibility at suitable concentrations, laying the groundwork for further investigation of their bioactivity in cellular environments.

Understanding the metabolic characteristics of UM is crucial for addressing radiotherapy resistance. We performed metabolomic assays on normal retinal pigment epithelial cells (ARPE19) and UM cells (MEL290, MUM2B, and OMM2.3, 92.1). Lactic acid levels are a common feature among the UM cell lines, suggesting a conserved metabolic shift in these cancer cells (Figure S10, Supporting Information). Therefore, targeting elevated lactic acid levels may provide a potential strategy for metabolic sensitization of radiotherapy. After co‐incubation with cells, the LDO nanosheets demonstrated exceptional capabilities in clearing lactic acid while simultaneously increasing the levels of pyruvic acid. The unique capability of LDO was further corroborated by mitigating lactic acid build‐up typically induced by radiotherapy (Figure 4c).

To assess intracellular ROS levels, dichlorodihydrofluorescein diacetate (DCFH‐DA) fluorescence imaging was performed in cells exposed to the LDO nanosheets (Figure 4d). While the control group showed minimal green fluorescence, indicative of low ROS levels, cells treated with LDO exhibited enhanced fluorescence, indicating elevated ROS levels. These data support the notion that LDO possesses intrinsic peroxidase‐mimicking activity, potentially increasing intracellular ROS levels. Moreover, by introducing 50 µg mL^−1^ LDO to UM cells, we detected increased generation of •OH after radiotherapy, thereby emphasizing LDO‐induced ROS promotion properties during radiotherapy. Furthermore, we observed a significant increase in the red fluorescence signal from the [Ru(dpp)_3_]Cl_2_ oxygen probe in cancer cells treated with LDO, which served as a strong indicator of intracellular O_2_ generation. The [(Ru(dpp)_3_)]Cl_2_ staining assay revealed marked alleviation of hypoxic conditions in the LDO‐treated group (Figure 4e).

Considering the potential of using LDO in vivo, we have also measured the hydrodynamic size of LDO in water, PBS, and DMEM over the course of one week to assess both size dynamics and stability (Figure S11, Supporting Information). Our results show that the hydrodynamic size of LDO increases over time in all three mediums, likely due to aggregation. In PBS, the hydrodynamic size was slightly larger than in the other two liquids, which could be attributed to the intercalation of the abundant anions in PBS into the structure of LDO, leading to its expansion. Regarding stability, LDO exhibited the best stability in water, with a slight increase in stability over time observed in PBS and DMEM.

### 2.5 In Vitro Radiosensitization Efficacy of LDO Nanosheets

We have outlined the multifaceted roles of LDO nanosheets in UM cells: clearance of lactic acid, amplification of ROS, and generation of oxygen. These cornerstone functionalities are purposefully engineered to enhance the effectiveness of radiotherapy, especially the lactic acid‐scavenging properties. UM cells incubated with lactic acid showed pronounced resistance to radiotherapy (Figure S12, Supporting Information), suggesting that high lactic acid levels in UM cells contribute to radioresistance. This observation offers the intriguing possibility that targeting lactic acid metabolism could be a promising strategy to overcome resistance to radiotherapy in UM. In the following stage, it is logical to examine the radiosensitization effect of LDO.

**Figure 5:**
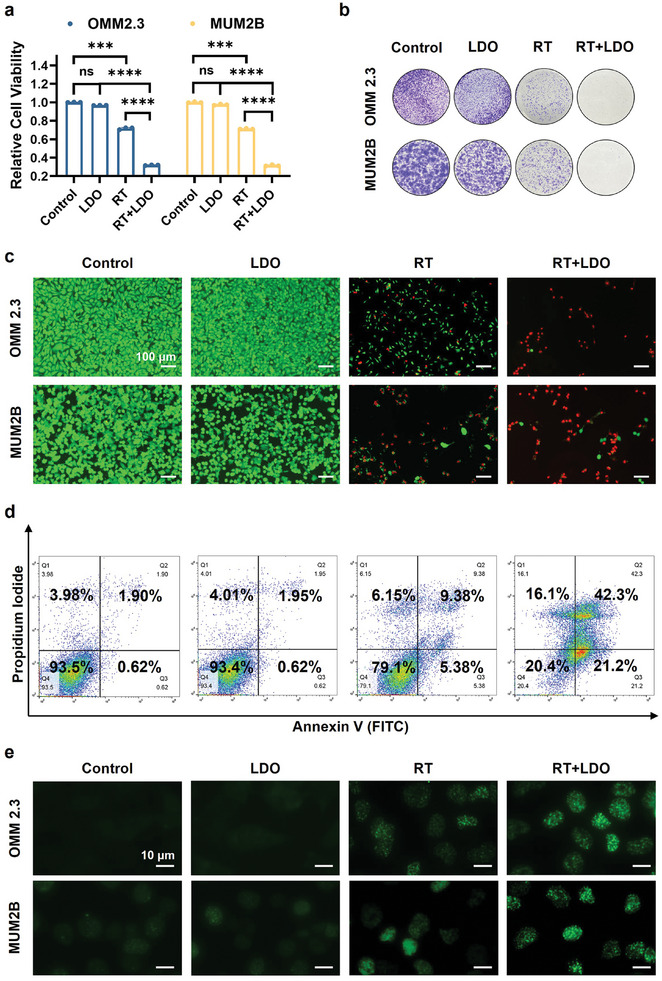
In vitro radiosensitization efficacy of LDO nanosheets. The radiosensitizing effect of LDO was determined using multiple assays. Four experimental conditions were compared: Control, LDO nanosheet treatment alone, radiotherapy alone, and a combination of LDO nanosheets and radiotherapy (RT). Three biological replicates were considered. a) Cellular viability quantified using a CCK8 assay. Data represent three biological replicates. b) Colony formation assay. c) Live/dead staining assay to determine cell viability. Scale bar: 100 µm. d) Flow cytometric analysis of Annexin V‐and propidium iodide‐stained cells. e) Immunostaining for γ‐H2AX across experimental groups. Scale bar: 10 µm.

A rigorous set of laboratory experiments confirmed the ability of LDO nanosheets to improve the effectiveness of radiation treatment on UM cells (OMM2.3 and MUM2B cells). The viability of UM cells (OMM2.3 and MUM2B), as measured by the CCK8 assay, demonstrated that the combined application of LDO and radiotherapy significantly reduced cell survival compared to either LDO or radiotherapy alone (**Figure** **5**a). This effect was further supported by a colony formation assay, which showed a considerable decrease in the long‐term survival of UM cells with combination treatment (Figure 5b). Live/dead cell staining confirmed these results (Figure 5c). Flow cytometry analysis elucidated the elevated apoptosis of UM cells treated with LDO and radiotherapy (Figure 5d). The combined treatment of radiotherapy with LDO further decreases the mitochondrial membrane potential, as indicated by the JC‐1 staining results (Figure S13, Supporting Information). A reduction in Δψ serves as an indicator of mitochondrial health deterioration, which may also signify early stages of apoptosis.

To further estimate the radiotherapy sensitization ability of LDO, the radiobiological parameters of the multi‐target single‐hit model were calculated (Figure S14, Supporting Information). The extrapolation number (N) for the control group and LDO group are 8.307 and 7.273, respectively. The mean lethal doses (D_0_) are 1.139 and 0.986. The radiation dose needed for a survival rate of 37% (D_37_) are 3.551 and 2.943. The sensitizer enhancement ratio (SER) values is 1.155.

Mechanistically, the increased effectiveness of the combined treatment could be attributed to the increased DNA damage in cells. This was verified by γ‐H2AX immunostaining, a well‐known marker for DNA double‐strand breaks, which indicated a significant increase in cell DNA damage under the combined treatment regime (Figure 5e). Increased DNA damage may be induced by enhanced lactic acid scavenging and ROS generation, alleviation of hypoxia, or decreased lactic acid levels.

Taken together, these data strongly suggest that LDO nanosheets enhance the effects of radiation therapy through the mechanisms mentioned in Section 2.4, making LDO an exciting candidate for improving radiation treatment, particularly in cases resistant to conventional therapy. These findings warrant exploration of the applicability of LDO across a broader spectrum of cancer types. To further validate its radiosensitizing effects, we extended our study to include human glioblastoma cells (U251) and human lung adenocarcinoma cells (A549), which represent a diverse array of cancers that could benefit from enhanced radiotherapy outcomes (Figure S15, Supporting Information). The combined application of LDO and radiotherapy significantly reduced cell survival, suggesting that LDO holds promise for enhancing radiotherapy across a wide variety of cancer types.

### 2.6 LDO Nanosheets as a Radiosensitizing Agent in an Orthotopic UM Model

**Figure 6:**
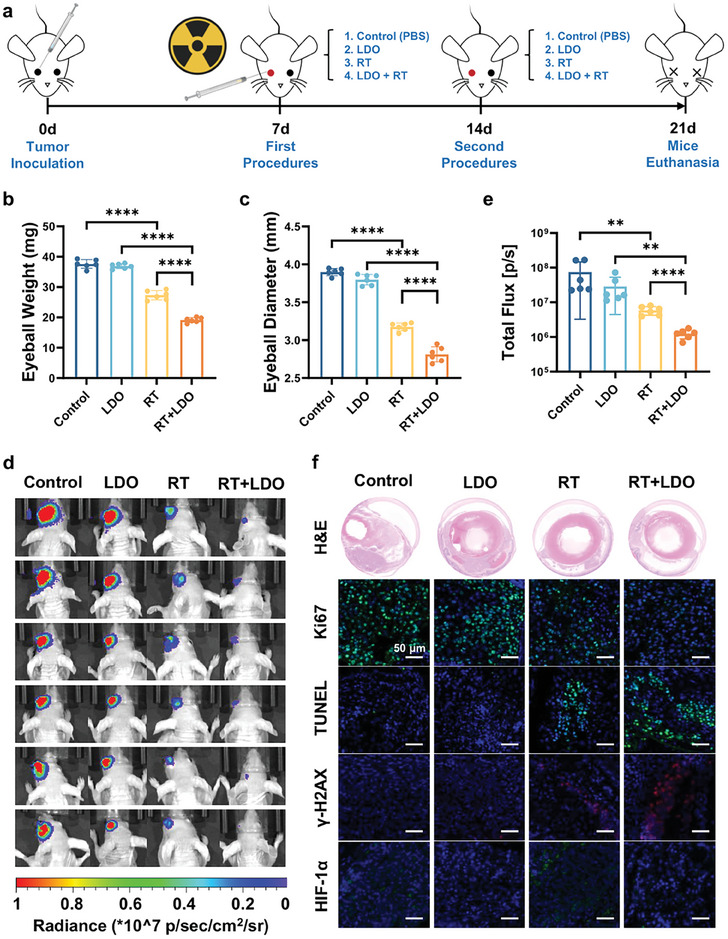
Evaluation of LDO nanosheets as radiosensitizing agents in an orthotopic UM model. a) Schematic overview of the experimental design used to test the radiosensitization ability of LDO in orthotopic UM models. b) Comparative weight measurements of eyeballs across four experimental groups: control; LDO nanosheet monotherapy; radiotherapy monotherapy; and LDO nanosheets combined with radiotherapy. c) Differential eyeball diameter measurements in the four treatment groups. d) In vivo fluorescence imaging of mice at the 2‐week post‐treatment with milestone. e) Quantification of the fluorescence intensity across groups. In results b–e), data represent three biological replicates. f) Histological and immunohistochemical evaluations of the eyeballs via hematoxylin and eosin (H&E), Ki67, terminal deoxynucleotidyl transferase dUTP nick end labeling (TUNEL), γ‐H2AX, and hypoxia‐inducible factor 1‐alpha (HIF‐1α) staining across different treatment groups. Representative images of three experimental replicates are shown. Scale bar: 50 µm.

The radiosensitization efficacy of the LDO nanosheets was further assessed in an orthotopic UM model, specifically using OMM2.3 cells injected into the eyes of mice. Treatment began on day 7, with four treatment groups: a control group receiving sterile phosphate‐buffered saline (PBS), a group treated with LDO nanosheets, a group undergoing radiotherapy, and a group receiving both LDO and radiotherapy. Treatments were repeated on day 14, and thorough analyses were conducted seven days after the final treatment (**Figure** **6**a).

Among the four groups, the group that received a combination of LDO nanosheets and radiotherapy exhibited the strongest tumor inhibitory capacity, as evidenced by the lowest eyeball weight and smallest diameter (Figure 6b,c; Figure S16, Supporting Information). Moreover, animal fluorescence imaging further validated the robust antitumor effects of the combined treatment, revealing reduced bioluminescence and smaller tumor volumes (Figure 6d–e). To further understand the effects on the TME, histological examinations were performed (Figure 6f). Hematoxylin and eosin (H&E) staining confirmed that LDO significantly enhanced the antitumor effects of radiotherapy in UM. Immunofluorescence assays using various markers showed multi‐pronged effectiveness: Ki67 staining, terminal deoxynucleotidyl transferase dUTP nick‐end labeling (TUNEL), γ‐H2AX, and hypoxia‐inducible factor 1‐alpha (HIF‐1α). The combination therapy resulted in decreased cellular proliferation, as indicated by diminished Ki67 staining. The combination treatment also increased apoptosis, as demonstrated by elevated TUNEL staining. Moreover, combination treatment amplified DNA damage, as shown by escalated γ‐H2AX staining, a classic indicator of DNA double‐strand breaks. Finally, the combination therapy alleviated hypoxia, as indicated by reduced HIF‐1α staining, which is a well‐recognized marker of the cellular response to hypoxia. Metabolic examination of subcutaneous tumors in nude mice further corroborated the unique capability of LDO to attenuate the lactic acid elevation typically induced by radiotherapy (Figure S17, Supporting Information), indicating that a critical mechanism underlies the radiosensitizing effects of LDO. These results provide robust evidence that LDO nanosheets can effectively potentiate the radiosensitizing effect of UM, which could be attributed to their three roles: mitigating hypoxia, producing ROS, and enhancing lactic acid scavenging. Thus, LDO nanosheets have emerged as a novel and promising therapeutic modality for the radiosensitization of UM.

Accumulating evidence suggests that lactic acid in the tumor microenvironment can modulate the metabolism of innate and adaptive immune cells.^[^ ^17^, ^23^ ^]^ Our findings indicate that LDO possesses inherent immune stimulatory activity, evidenced by an increase in CD8^+^T lymphocyte infiltration (Figure S18, Supporting Information). This effect is potentially related to LDO's ability to clear lactic acid. Suppressing the number and activity of CD8^+^T cells, thereby inhibiting antitumor immunity. LDO, by reducing lactic acid levels, may reverse the lactic acid‐induced suppression of CD8^+^T cells. Furthermore, radiotherapy enhances the immunogenicity of tumor cells and the antitumor immune response,^[^ ^24^ ^]^ as observed in the radiotherapy group. It has been reported that radiotherapy can reshape the immune microenvironment through a variety of mechanisms, including inducing immunogenic death and antigen release of tumor cells, inducing T‐cell proliferation and activation, and improving T‐cell homing and infiltration to tumors.^[^ ^25^ ^]^ The combined LDO and radiotherapy group exhibited the highest infiltration of CD8^+^T lymphocytes, attributable both to the reduction of lactic acid‐induced immune suppression and the increased immunogenicity induced by radiotherapy. This enhanced antitumor immunity could contribute significantly to the therapeutic efficacy of combined treatments in potential clinical applications.

We propose a novel radiosensitizer, LDO, based on a crucial obstacle in the treatment of UM, which is the confounding role of lactic acid in fostering resistance to radiotherapy.^[^ ^15a^ ^]^ Current strategies aimed at managing lactic acid levels have limitations, underlining the pressing need for increased effectiveness. One strategy employs small‐molecule inhibitors that focus on lactate‐producing enzymes and transporters, specifically lactate dehydrogenase A (LDHA) and monocarboxylate transporter 1 (MCT1).^[^ ^26^ ^]^ Although LDHA is key to converting pyruvic acid into lactic acid, inhibitors targeting it have limitations; they do not effectively address the excess lactic acid that accumulates after radiotherapy.^[^ ^13b^ ^]^ Similarly, MCT1 is essential for lactate transport; however, its inhibition often leads to resistance issues.^[^ ^27^ ^]^ When MCT1 is successfully inhibited, other lactate transport pathways can take over, negating the therapeutic benefits.^[^ ^27^ ^]^ More critically, these strategies fail to remove accumulated lactate from the TME, which continues to facilitate tumor progression and resistance to therapy.^[^ ^28^ ^]^ Nanomaterials have emerged as potential game changers for effective lactate clearance. Basic nanoparticles, such as bicarbonate and calcium carbonate, have been employed to neutralize the acidity of TME.^[^ ^29^ ^]^ However, such neutralization approaches are intrinsically limited; they fail to fully address the non‐acidic mechanisms by which lactic acid promotes tumor growth and resistance to treatment.^[^ ^23^ ^]^ For example, simply adding hydrochloric acid does not recreate the complex or malignant effects of lactic acid, demonstrating that neutralizing acidity alone is insufficient.^[^ ^12^, ^23^ ^]^

A more recent innovation involves nanomaterials encapsulating lactate oxidase, which shows considerable promise for sensitizing chemotherapy, altering the immune TME, and enhancing starvation therapy.^[^ ^30^ ^]^ However, these approaches are not without challenges, including the high costs of synthesis, purification, and stability concerns in complex biological environments.^[^ ^18^ ^]^ Rather than offering minor improvements, our research fundamentally reconfigures the understanding and tackling of lactate‐induced resistance to radiotherapy in patients with UM. In this study, we introduced LDO nanosheets as a groundbreaking solution with unprecedented lactate‐clearing capabilities, thereby sensitizing tumors to radiotherapy. By focusing on the complex metabolic mechanisms of radiotherapy resistance in UM, we provide evidence that this nanomaterial could represent a transformative treatment modality. Our computational analyses further illuminated the lactate‐clearing potential of LDO nanozymes, setting the stage for their application in biological systems. The synthesis of models and experimentation strengthens the impact of this study, making it a crucial contribution to the fields of radiosensitization and nanozymes. Thus, our study unveiled a pioneering approach to overcome the challenges of radiotherapy sensitization in UM. LDO nanosheets are a beacon of hope in the ongoing quest to overcome the clinical hurdles associated with UM treatment. This discovery may extend beyond UM, heralding a new era of precision medicine and targeted therapeutic strategies.

## 3 Conclusion

UM presents a unique therapeutic challenge due to its radiation resistance, which is largely attributed to factors like elevated lactic acid levels, hypoxic TME, and diminished ROS production. This study introduces an innovative solution with the synthesis of LDO nanosheets. These LDO nanosheets showcase efficient lactic acid scavenging, alongside enhanced ROS production and oxygen generation. They catalyze the transformation of hydrogen peroxide into more toxic hydroxyl radicals, thereby significantly amplifying ROS production during radiotherapy. Concurrently, LDO efficiently scavenged lactic acid, thereby impeding the DNA and protein repair in tumor cells, thus synergistically intensifying radiotherapy's impact. The in vitro analyses reveal LDO's favorable biocompatibility and minimal toxicity. As a potent radiosensitizer, LDO demonstrates a remarkable ability to destroy uveal melanoma cells at a 6 Gy radiotherapy dose. In vivo studies further confirm LDO's substantial tumor inhibitory effect in an orthotopic UM model under a total radiotherapy dose of 12 Gy. This research positions LDO nanosheets as highly active, responsive to the tumor microenvironment, and enduring nanomaterials, significantly enhancing radiotherapy sensitization in UM. The novel LDO nanozyme holds promise for improving radiotherapy outcomes across a wide spectrum of cancers, including UM.

## Conflict of Interest

The authors declare no conflict of interest.

## Supporting information

Supporting Information

## Data Availability

The data that support the findings of this study are available from the corresponding author upon reasonable request.
