# Supplementary material for: A Novel Nanozyme to Enhance Radiotherapy Effects by Lactic Acid Scavenging, ROS Generation, and Hypoxia Mitigation
Source: Adv Sci (Weinh). 2024 May 5;11(26):2403107. doi: 10.1002/advs.202403107 (PMC11234405; doi:10.1002/advs.202403107)
Supplement: Supplementary file 1 — Supporting Information [file ADVS-11-2403107-s001.docx]

Supporting Information

A Novel Nanozyme to Enhance Radiotherapy Effects by Lactic Acid Scavenging, ROS Generation, and Hypoxia Mitigation

Yiran Yao, Ru Xu, Weihuan Shao, Ji Tan*, Shaoyun Wang, Shuhan Chen, Ai Zhuang*, Xuanyong Liu*, and Renbing Jia*

Experimental Section

*Material Preparation*: Cobalt nitrate hexahydrate (Co(NO_3_)_2_·6H_2_O), iron nitrate nonahydrate (Fe(NO_3_)_3_·9H_2_O), manganese chloride tetrahydrate (MnCl_2_·4H_2_O), sodium hydroxide (NaOH), sodium bicarbonate (NaHCO_3_), and 30% hydrogen peroxide solution were procured from Sinopharm Chemical Reagent, China. 3,3′,5,5′-tetramethylbenzidine (TMB) and methylene blue were sourced from Sigma-Aldrich (USA).

*LDO Nanosheets Synthesis*: A mixture of 0.44 g Co(NO_3_)_2_·6H_2_O, 0.15 g MnCl_2_·4H_2_O, and 0.30 g Fe(NO_3_)_3_·9H_2_O was prepared in 50 mL of deionized water. Subsequently, 3 mL of 1 M NaHCO_3_ was added and the pH of the solution was adjusted to 10 using 10 M (400 μL) NaOH, followed by magnetic stirring for 30 minutes. After centrifugation, the resulting precipitate was rinsed with ultrapure water and ethanol to yield a compound designated LDH. The transformation into LDO samples was accomplished by calcining the LDH at 250 °C for 5 hours.

*LDO Characterization*: The sample morphologies were analyzed using TEM (Tecnai G2 F20, Netherlands). XRD (D/Max, Rigaku, Tokyo, Japan) was used to determine the phase composition of the LDO. N_2_ adsorption–desorption isotherms were obtained at 77 K using an Autosorb-1 surface area analyzer (Quantachrome). An ASAP 2460 surface area and porosity analyzer (Micromeritics, USA) was used to determine the specific surface areas of the samples.

*TMB Oxidation Assay*: The assay was performed using sodium acetate-acetic acid (NaAc-HAc) buffer solutions at various pH levels, 2 mM 3,3',5,5'-tetramethylbenzidine (TMB) as the chromogenic substrate, 0.01 M hydrogen peroxide solution, and LDO dispersion. In a 96-well plate, 10 μL of LDO dispersion, 10 μL of TMB solution, 10 μL of hydrogen peroxide, and 70 μL of NaAc-HAc buffer were added to each well. The reaction was carried out at a predetermined temperature. The ultraviolet absorption spectra (500-800 nm) were measured after 20 minutes using a multifunctional microplate reader (Cytation 5, BIOTEK, USA).

*Peroxidase-like Activity Kinetics Evaluation via TMB Oxidation Assay:* The enzymatic behavior (peroxidase-like activities) of LDO was investigated through a TMB oxidation assay, employing sodium acetate-acetic acid (NaAc-HAc) buffer solutions at a pH of 4, 2 mM 3,3',5,5'-tetramethylbenzidine (TMB) as the chromogenic substrate, and hydrogen peroxide solutions at varying concentrations (0 mM, 0.01 mM, 0.1 mM, 1 mM, 10 mM). A 100 μg/mL concentration of LDO dispersion was used for the assay. Each reaction setup in a 96-well plate included 10 μL of the LDO dispersion, 10 μL of TMB solution, 10 μL of hydrogen peroxide solution, and 70 μL of the NaAc-HAc buffer. Reactions were incubated at 37°C. Ultraviolet absorption at 652 nm was monitored at predetermined time intervals (0, 1, 2, 3, ..., 10 minutes) using a multifunctional microplate reader (Cytation 5, BIOTEK, USA). The reaction rate (v) at various concentrations of hydrogen peroxide was determined from the initial slope of hydrogen peroxide consumption. These reaction rates were then plotted against the hydrogen peroxide concentrations and subjected to Michaelis-Menten curve fitting to extract the Michaelis-Menten constant (K_m_) and the maximum initial velocity (V_max_). The enzyme activity was also examined under various parameters such as different concentrations, pH values and temperatures.

*DHE Fluorescence Assay*: Assays were conducted by preparing sodium acetate-acetic acid (NaAc-HAc) buffer solutions with varying pH levels, 1 μg/mL dihydroethidium (DHE) solution, 0.01 M hydrogen peroxide solution, and LDO dispersion. In a 96-well plate, 10 μL of the prepared LDO dispersion, 10 μL of DHE solution, 10 μL of hydrogen peroxide, and 70 μL of NaAc-HAc buffer were added to each well. The reaction mixture was incubated at a set temperature. The fluorescence spectra (380-500 nm) were measured after 20 minutes using a multifunctional microplate reader (Cytation 5, BIOTEK, USA).

*Catalase-Like Activity Assay of LDO*: The catalase-like activity of LDO was quantitatively assessed by measuring the oxygen generation from the catalytic decomposition of hydrogen peroxide. The assay was conducted using sodium acetate-acetic acid (NaAc-HAc) buffer solutions at a pH of 6, with hydrogen peroxide solutions prepared at varying concentrations (0 mM, 0.01 mM, 0.1 mM, 1 mM, 10 mM). For this purpose, a dispersion of LDO at a concentration of 50 μg/mL was utilized. The reactions were performed at a constant temperature of 24°C. The generation of dissolved oxygen was monitored over time at specific intervals (0, 5, 10, 15, ..., 60 seconds) employing a dissolved oxygen meter (CH-9100, Herisau, Switzerland). The initial rate of oxygen generation (v) at different hydrogen peroxide concentrations was calculated based on the initial slope of the oxygen generation curve. Subsequently, these rates were plotted against the hydrogen peroxide concentrations for Michaelis-Menten curve fitting, allowing the determination of the Michaelis-Menten constant (K_m_) and the maximum initial velocity (V_max_). The enzyme activity was also examined under various parameters such as different concentrations, pH values and temperatures.

*TMB Oxidation Assay at different PH*: To assess peroxidase-mimic activity, an aqueous solution (pH = 6.5 or 7.4) containing LDO (50 μg/mL) was formulated. A 96-well plate was sequentially populated with 10 μL each of the sample, H_2_O_2_, and 20 mM TMB solutions, along with 70 μL of NaAc-HAc buffer (pH = 6.5 or 7.4). Peroxidase-like kinetic characteristics were analyzed from 500 to 850 nm using a Cytation 5 microplate reader (BIOTEK, USA).

*Measurement of Oxygen Generation via Dissolved Oxygen Meter:* The generation of oxygen in the presence of layered double oxide (LDO) nanosheets was quantified using a precise dissolved oxygen assay. The assay utilized sodium acetate-acetic acid (NaAc-HAc) buffer solutions adjusted to pH values of 6.5 and 7.4, combined with hydrogen peroxide at a concentration of 100μM. For the experiment, an LDO dispersion was prepared at a concentration of 50 μg/mL. All reactions were carried out at a controlled temperature of 24°C. The temporal evolution of dissolved oxygen levels was systematically recorded at predetermined intervals (0, 30, 60, 90, ..., 330 seconds) using a dissolved oxygen meter (Model CH-9100, Herisau, Switzerland). This approach facilitated the accurate monitoring of oxygen generation dynamics in response to the catalytic activity of LDO under the specified experimental conditions.

*Detection of ·OH and ·O_2_^-^ Using Electron Spin Resonance (ESR) Assays*: The assay for ·OH radicals was performed in sodium acetate-acetic acid (NaAc-HAc) buffer solutions, pH 6.5, with a 50 mM concentration of hydrogen peroxide. A 500 μg/mL dispersion of LDO was prepared for the assay. The LDO dispersion was mixed with the hydrogen peroxide solution, and the reaction was allowed to proceed for 10 minutes before measurement of the radicals. A control experiment, lacking LDO, was conducted under identical conditions to assess the background radical generation. For the detection of ·O_2_^-^ radicals, ethanol was used as the solvent, with a 50 mM hydrogen peroxide concentration. Similarly, a 500 μg/mL LDO dispersion was prepared. The dispersion was mixed with the hydrogen peroxide solution, and the mixture was reacted for 10 minutes prior to the radical measurement. A blank control group without LDO was also tested under the same conditions to establish the baseline level of radical generation.

*Transformation of Lactic Acid:* To explore the possible influence of LDO on lactic acid production, lactic acid consumption and pyruvic acid production were monitored. Detection was accomplished using the LA assay kit (Solarbio® BC2230) and PA assay kit (Solarbio® BC2200) after incubation of LDO (50 µg/mL or 100 µg/mL) with a lactic acid solution (3 mM).

*Lactic Acid Depletion Kinetics Assay*: To evaluate the catalytic activity of LDO towards lactate oxidation, a reaction mixture containing 5 mM L-lactic acid and 50 μg/mL LDO was prepared in a total volume of 1 mL. The mixture was incubated under constant agitation at room temperature. At predetermined time intervals (0, 20, 40, ..., 100 minutes), samples were taken to assess the consumption of lactate. The quantification of lactate was performed using a lactate assay kit (Solarbio® BC2230) according to the manufacturer's instructions.

*DFT Calculations*: To further deepen our understanding, the Vienna Ab initio Simulation Package (VASP) was used to perform all DFT calculations. These calculations were performed within the framework of the generalized gradient approximation using the Perdew (Burke) Ernzerhof functional. Specifically, the projected augmented wave potentials were selected to accurately characterize the ionic cores and to consider the valence electrons using a plane-wave basis set with an energy threshold of 450 eV. Geometry optimizations were performed using stringent criteria to ensure a force convergence of less than 0.05 eV/Å. For all computational processes, 1 × 2 × 1 Monkhorst–Pack k-points were applied, and the influence of the spin polarization was considered. Additionally, the DFT-D3 empirical correction method was applied to accurately capture the van der Waals interactions, while the DFT+U approach was employed to specifically treat Mn, Fe, and Co in highly localized 3d states using the corresponding parameters of U–J = 3.9, 4.3, and 3.3 eV respectively. The vacancy formation energy was calculated using the following equation: ΔE=E(Ov)+1/2E(O_2_)-E(Surf), where E(Ov) is the total energy of the vacancy surface and E(O_2_) is the energy of O_2_.

*Gas Chromatography-Mass Spectrometry (GC-MS) Analysis for detecting lactic acid and pyruvic acid*: To complementarily assess the variations in lactate and pyruvate concentrations, gas chromatography-mass spectrometry (GC-MS) was employed as a non-quantitative method. Samples were centrifuged at 15,000 g for 10 minutes at 4°C, and the supernatants were collected. A 1 μL aliquot of the supernatant was used for GC-MS analysis. The chromatographic separation was conducted using water (containing 5 mM ammonium acetate) as mobile phase A and acetonitrile as mobile phase B. The flow rate was set at 0.30 mL/min, and the column temperature was maintained at 45°C. The Thermo HESI-II ion source was operated in selected reaction monitoring (SRM) mode to enhance the specificity of the detection of lactic acid and pyruvic acid.

*Cell Culture*: The human UM cell line MUM2B was cultivated in DMEM (GIBCO, USA) with supplements and maintained at 37 °C in a humidified 5% CO_2_ environment. The adult retinal pigment epithelial cell line-19 (ARPE19) and human UM cell lines OMM2.3 were similarly cultured in RPMI 1640 medium (GIBCO, USA) with identical supplements and conditions.

*CCK8*: To evaluate the viability of cells in all experimental groups (Control, LDO, Radiotherapy, LDO+ Radiotherapy) in vitro, a CCK8 assay (NCM, Suzhou, China) was performed. The LDO concentration was 50 µg/mL. ARPE-19, OMM2.3, and MUM2B cells (3×10^4^) were seeded into 24-well plates and incubated overnight with LDO or PBS. All samples were transferred to plates and the two groups were subjected to ionizing radiation at a dose of 6 Gy. The cells were treated with CCK8 kits and the results were measured.

*Evaluation of Intracellular ROS Levels*: Cells (density: 1.0 × 10^5 cells/well) were seeded in 24-well plates for 24 hours and treated with all experimental groups (Control, LDO, Radiotherapy, LDO+ Radiotherapy). After a 24-hour incubation, DCFH-DA solution was added, followed by 30 minutes of culture. Fluorescence images were captured using a microscope to assess intracellular ROS levels.

*Oxygen Generation Investigation via [(Ru(dpp)_3_)]Cl_2_ Staining*: Oxygen production in tumor cells was examined using the O_2_-sensitive probe [(Ru(dpp)_3_)]Cl_2_. The fluorescence of the reagent was strongly quenched by oxygen. Cells were cultured (density: 5 × 10^3 cells/well) for 24 hours, with or without LDO. All samples were transferred to plates and the two groups were subjected to ionizing radiation at a dose of 6 Gy. Following a 4-h incubation with [(Ru(dpp)_3_)]Cl_2_, the fluorescence signal (E_x_=488 nm, E_m_=610 nm) was visualized and documented using a confocal laser scanning microscope (Olympus, Japan).

*Cell Viability Assessment through Live/Dead Staining*: To evaluate the viability of cells in all experimental groups (Control, LDO, Radiotherapy, LDO + Radiotherapy) in vitro, a live/dead staining assay was performed. The concentration of LDO was 50 µg/mL. A total of 3×10^4 ARPE-19, OMM2.3, and MUM2B cells were seeded into 24-well plates and incubated overnight with LDO or PBS. All samples were transferred to plates and the two groups were subjected to ionizing radiation at a dose of 6 Gy. Cells were stained using a live/dead staining kit and analyzed using a confocal laser scanning microscope.

*JC-1 Staining*: For mitochondrial membrane potential analysis using JC-1 staining, cells were cultured at a density of 5 × 10^3 cells/well for 24 hours, with or without the addition of LDO. Subsequently, all samples were exposed to ionizing radiation at a dose of 6 Gy. Post-radiation, the cells were stained with JC-1 probe according to the manufacturer's recommended protocol (Mitochondrial Membrane Potential Assay Kit (JC-1), Beyotime). For the detection of JC-1 monomers, the excitation and emission wavelengths were set at 490 nm and 530 nm, respectively. For JC-1 aggregates, the excitation was set at 525 nm with emission at 590 nm. Imaging and documentation were performed using a confocal laser scanning microscope (Olympus, Japan).

*Flow Cytometry Assay*: The antitumor efficacy of various samples (Control, LDO, Radiotherapy, LDO+ Radiotherapy) was assessed in vitro by flow cytometry using 50 µg/mL LDO. OMM2.3 and MUM2B cells (3×10^4^) were cultured in 24-well plates overnight. Following digestion with 0.25% trypsin (without EDTA) and washing with PBS, apoptosis was identified by double staining with Annexin V-FITC and propidium iodide (BD Biosciences, USA). Apoptotic cells were quantified using a flow cytometer (FACSCalibur, BD Biosciences, USA) and analyzed using the FlowJo software.

*Colony Formation Assay*: UM cells (500 cells/well) were cultured in 12-well plates (Corning) and incubated at 37 °C for 1–2 weeks. After incubation, cells were rinsed with PBS, fixed with methanol, and stained with 1% crystal violet for 30 minutes. The excess dye was rinsed, and the colonies were air-dried, scanned using a Hewlett-Packard flatbed scanner, and quantified using ImageJ software (version 1.8.0, NIH, Bethesda, MD, USA).

*Multi-target Single-hit Model*: Uveal melanoma cells in the logarithmic growth phase were taken out and digested by trypsin to make single-cell suspension, and the cells were counted. Four dose points were set: 0Gy, 3Gy, and 6Gy, and 500 cells were inoculated into six-well plates, with three compound wells set for each dose group. Cells were cultured for 24 hours, with or without LDO After the cells were attached to the wall, the predetermined irradiation dose was given to the cells, and then the cells were placed in an incubator for static culture, and the cells were treated with liquid change according to the cell condition. After continuous culture for 14 days, crystal violet staining was used to observe the monoclonal number. The survival curves of cells were fitted by the formula Y=1-(1-EXP(-K*X))^N of the multi-target single-hit model, and the radiobiological parameters were calculated.

*Immunofluorescence*: Deparaffinized and rehydrated samples were fixed, blocked with 5% normal goat serum, and incubated with the primary antibody (1:400, ab15580, Abcam) at 4 °C overnight. After a 60-minute incubation with secondary antibodies, nuclei were counterstained with DAPI (Sigma-Aldrich, St. Louis, MO, USA) for 5 minutes. Digital images were captured using a ZEISS Axio Scope A1 upright microscope (Oberkochen, Baden-Württemberg, Germany).

*Metabolomics*: Cell samples and animal tissues were treated and harvested, followed by intracellular metabolite extraction using an 80% v/v aqueous methanol solution. The extract was then subjected to ultrasonication, centrifugation, and evaporation under gentle nitrogen stream for derivatization. Standard solutions were mixed, serially diluted, and combined with a quality control sample and dulcitol before drying under nitrogen. The analysis was performed using an Agilent 7890A gas chromatography system paired with an Agilent 5975C inert MSD system employing specific columns, helium carrier gas, and controlled temperatures for separation. Data collection was performed in designated scan modes and quantification was performed using the MSD ChemStation.

*Measurement of Lactic Acid and Pyruvic Acid*: The production of lactic acid and pyruvic acid was analyzed in the culture media or cells using the respective assay kits, following the manufacturer's guidelines.

*Evaluation of In Vivo Antitumor Activity*: Animal experiments were conducted in accordance with the animal policies of Shanghai JiaoTong University. Orthotopic ocular tumor models were established in BALB/c nude mice (female, 5 weeks old) to assess the antitumor effects of the experimental groups. Tumors were constructed according to Shanghai Jiao Tong University’s animal policies through a subretinal injection of 2 × 10^5 OMM2.3 cells. After a week, mice were administered 2 μL of either sterile PBS (Control and Radiotherapy) or 100 µg/mL LDO (LDO and RT+LDO); two groups received a radiation dose of 6 Gy (RT and RT+LDO). A subsequent round of treatment was administered after a week, with sacrifice and collection of eyeballs occurring one week after the final treatment. The eyeball diameter and weight were measured, followed by fixation and paraffin embedding. Tissue sections (4 μm thick) were immunostained with antibodies (H&E, Ki-67, TUNEL, γ-H2AX, HIF-1α; Bioworld Technology, Nanjing, China) and examined under a Leica TE2000-S microscope. H&E staining was also applied to organs including the heart, liver, spleen, lung, kidney, and brain.

*Evaluation of In Vivo Immune Stimulatory Activity*: Female C57BL/6 mice, aged 6–8 weeks, were utilized to establish a subcutaneous uveal melanoma tumor model. The B16F10 melanoma cells, in logarithmic growth phase, were dissociated into a single-cell suspension using trypsin solution. The suspension was transferred to a 15 mL centrifuge tube and centrifuged at 1600 rpm for 5 minutes, after which the supernatant was discarded. The cells were then resuspended in 5 mL of phosphate-buffered saline (PBS), washed by centrifugation at 1600 rpm for 5 minutes, and the supernatant was again discarded. The cells were resuspended in 1 mL of PBS, and cell count was determined under a microscope. Based on the counting results, the cell concentration was adjusted to 5×10^6 cells/mL, and the suspension was transferred to a 1.5 mL Eppendorf (EP) tube. Prior to inoculation, mice were weighed to ensure a body weight of approximately 18-22 g. The hair on the right dorsal side was removed with a shaver to expose the skin, which was then disinfected with 75% alcohol. The cell suspension was homogenized by flicking the EP tube, and 100 μL of the suspension, containing 5×10^5 cells, was drawn into a 1.5 mL syringe. The needle was inserted subcutaneously with the bevel facing up, and the cell suspension was slowly injected. After injection, the needle was rotated and withdrawn, and the injection site was pressed with a disinfected cotton ball for 1-2 minutes. Tumor growth was closely monitored post-injection, with successful model establishment indicated by the presence of a black mass at the tumor site approximately on day 7. One week post-tumor establishment, mice received an intratumoral injection of 100 μL of either sterile PBS (for the Control and Radiotherapy groups) or 0.5 µg/μL LDO dispersion (for the LDO and RT+LDO groups). Two of the groups also received a local radiation dose of 6 Gy (RT and RT+LDO groups). A second round of treatment was administered one week later, and the mice were sacrificed one week after the final treatment for tumor collection and analysis.

*Bioluminescence Imaging for In Vivo Antitumor Efficacy Evaluation*: OMM2.3/Luc cells were injected into the eyeballs of mice, with subsequent treatments mirroring the above description. One week after the second treatment, 150 mg/kg firefly luciferin was administered, followed by anesthesia with 1% pentobarbital sodium solution 15 minutes later. Bioluminescence was quantified on a Lago X Optical Imaging Platform (Cold Spring Biotech Corp.; 20 s exposure), and the average signal intensities within a circular ROI were analyzed.

*Data Analysis*: Statistical evaluation was performed using GraphPad Prism 5 (San Diego, Carlifornia USA). Data are represented as the mean ± SD, with significant differences (P) analyzed using unpaired t-tests. Significance levels are denoted as follows: *p*<0.05 (^*^), *p*<0.01 (^**^), *p*<0.001 (^***^), and *p*<0.001 (^****^).

Results and discussion

Figure S1. The 3,3’,5,5’-tetramethylbenzidine (TMB) oxidation assay.

Experiments utilizing both TMB Oxidation Assay （**Figure S2a**）and DHE Fluorescence Assay（**Figure S2b**）, show that LDO under radiation conditions produces more hydroxyl radicals and superoxide anions compared to non-radiation conditions. This suggests that LDO has significant potential as a radiosensitizer.


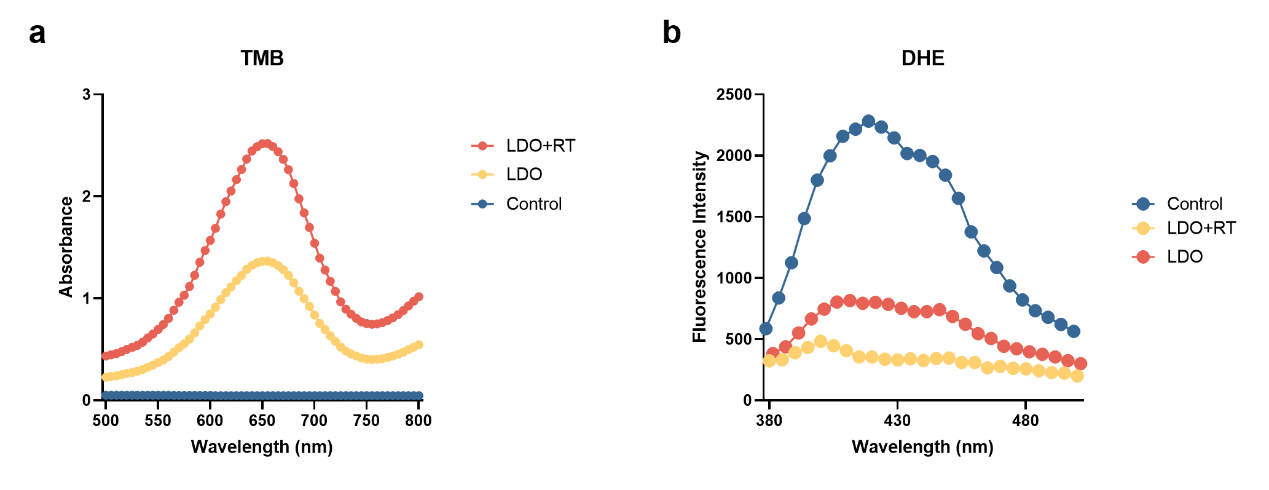


Figure S2. The ROS generation of LDO under radiation conditions. (a) TMB oxidation assay. (b) DHE fluorescence assay.

We found that the peroxidase-like activity of LDO strengthens continuously as the pH decreases from 7 to 3, indicating a pH-dependent behavior which is advantageous in the acidic tumor microenvironment for ROS generation (**Figure S3b**). Regarding temperature, the peroxidase-like activity of LDO increased and peaked as the temperature rose from 4 °C to 37 °C, with no significant increase in catalytic activity beyond this temperature (**Figure S3b**).


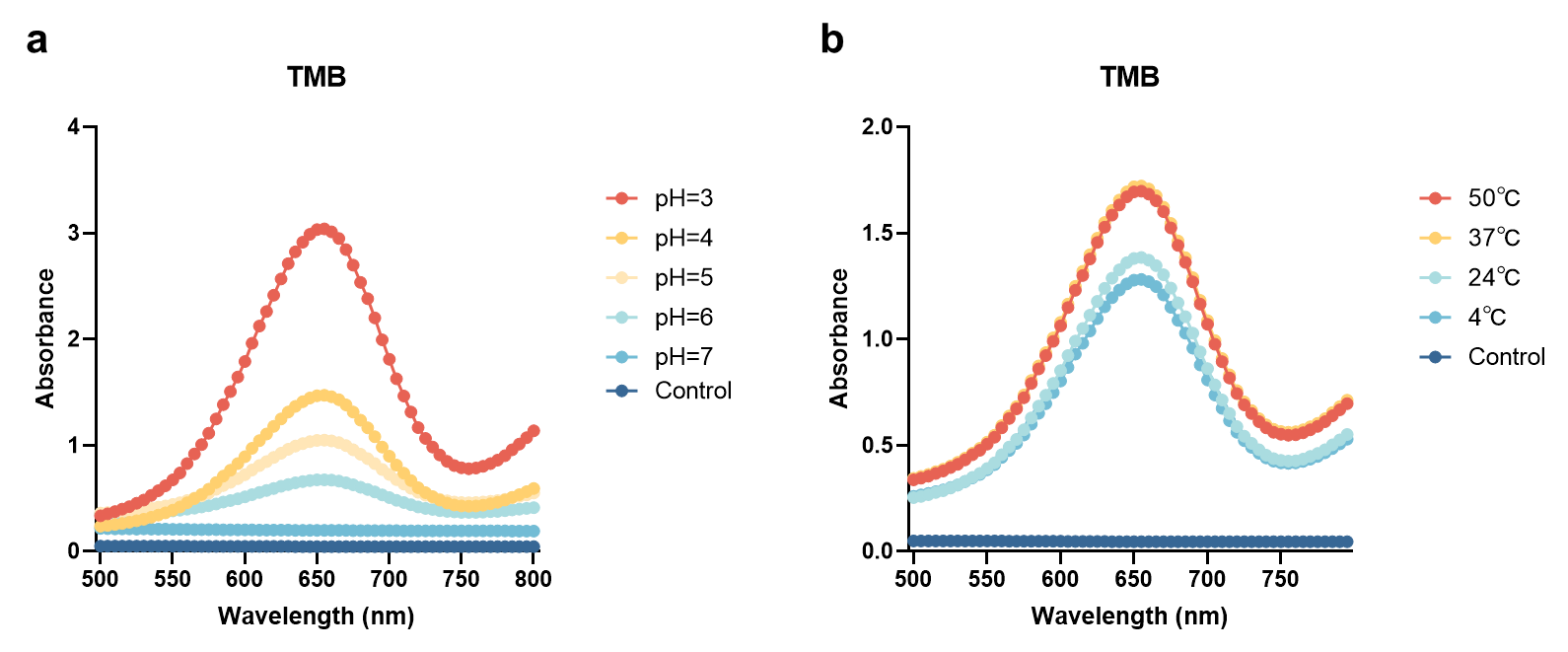


Figure S3. The relationship between peroxidase-like activity and parameters. (a) pH gradient. (b) temperature gradient.

We observed that the catalase-like activity of LDO increases with decreasing pH, from 7 to 3, indicating acid-dependent enzyme activity. Additionally, the activity enhances as temperature rises from 4 °C to 37 °C (**Figure S4a**). Furthermore, the relationship between enzyme activity and various parameters have been examined (**Figure S4b**).


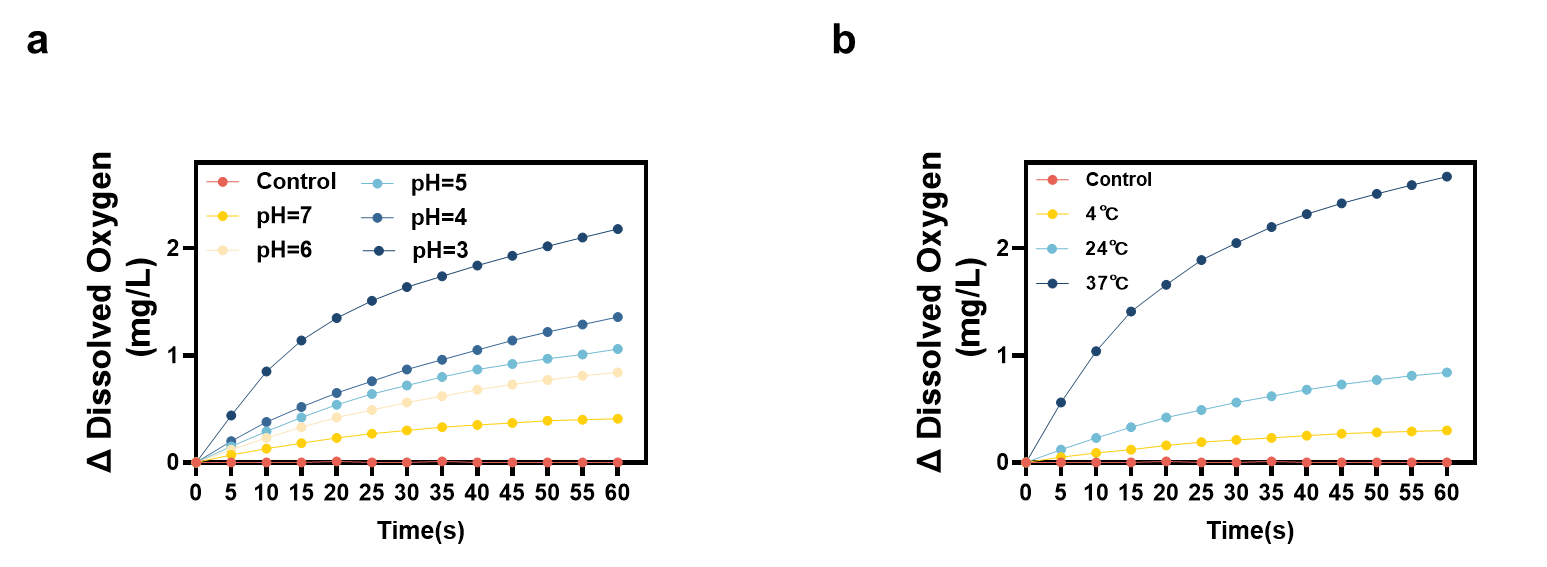


Figure S4. The relationship between peroxidase-like activity and parameters. (a) pH gradient. (b) temperature gradient.

Various oxygen vacancy formation scenarios based on this simulation model were considered in **Figure S5a**, including the vacancies formed at FeFeMn-O (**Figure S5c**), FeCoMn-O (**Figure S5d**), and CoCoMn-O (**Figure S5e**) sites. A simulation model with FeFeMn-O vacancies was selected for subsequent calculations because it exhibited the lowest vacancy formation energy (**Figure S5b**).


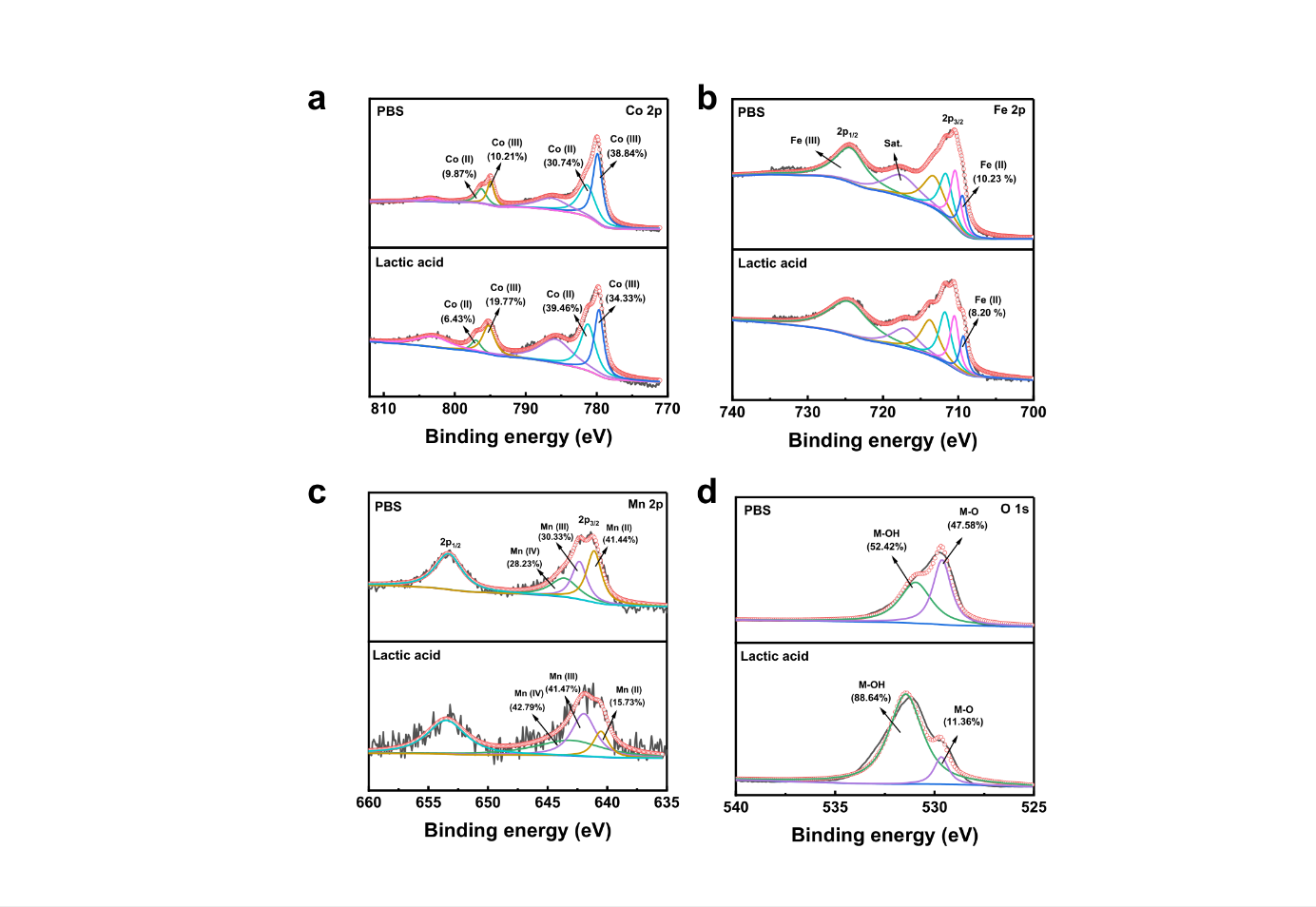


Figure S5. High-resolution spectra of Co (a), Fe (b), Mn (c), and O (d) in LDO immersed in PBS and lactic acid solution. *Abbreviations: PBS: phosphate-buffered saline*


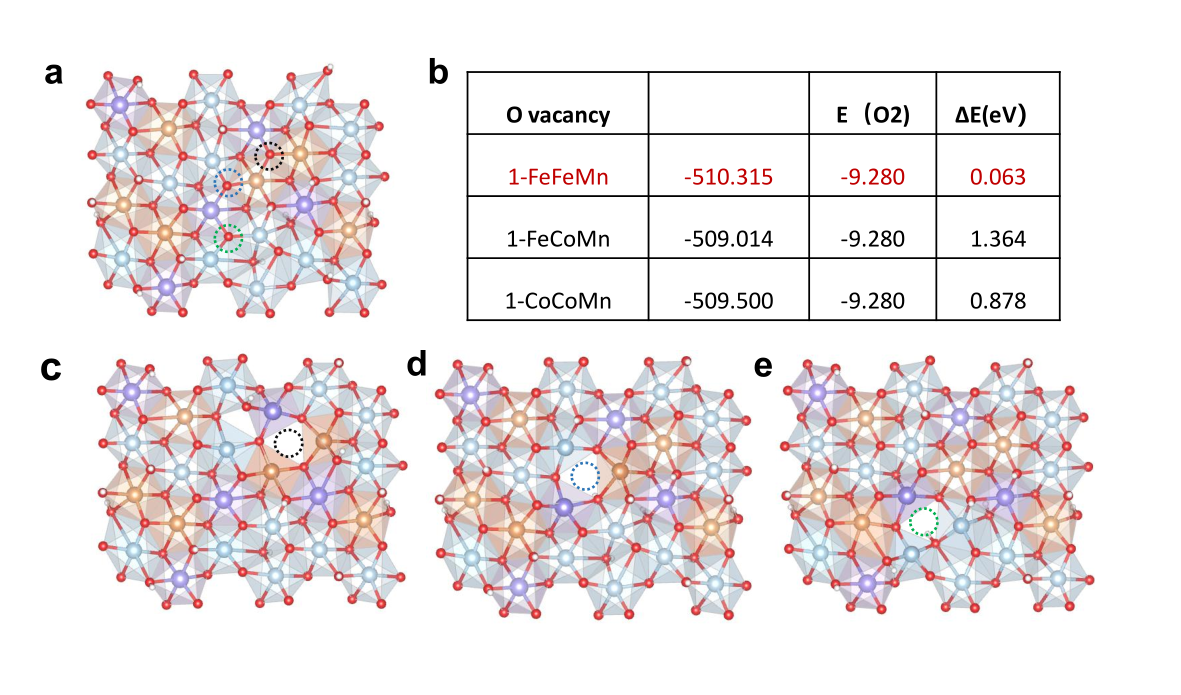


Figure S6. Various oxygen vacancy formation scenarios based on this simulation model.


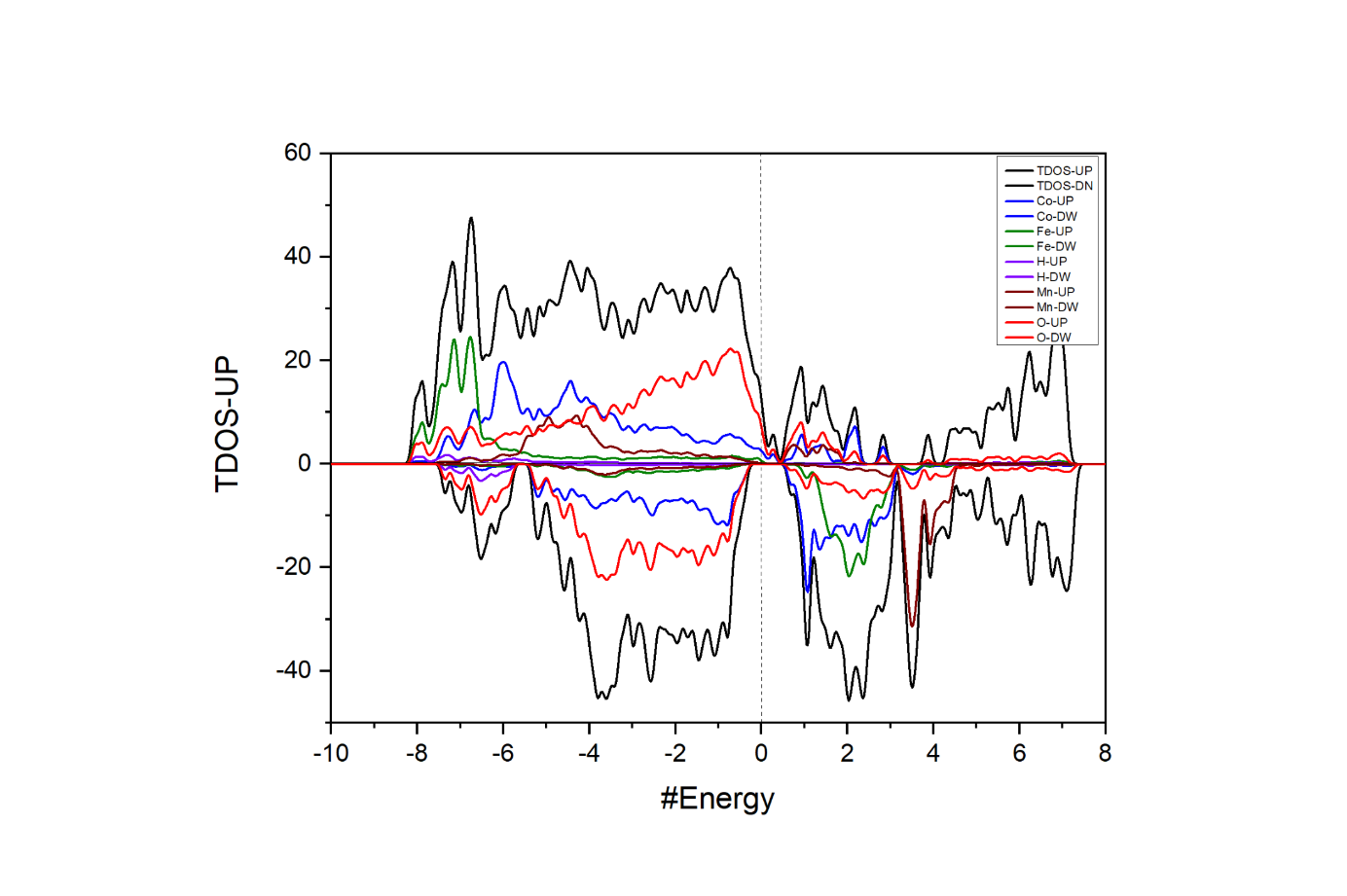


Figure S7. Calculated density of states of the LDO model. *Abbreviations: TDOS: total density of states.*


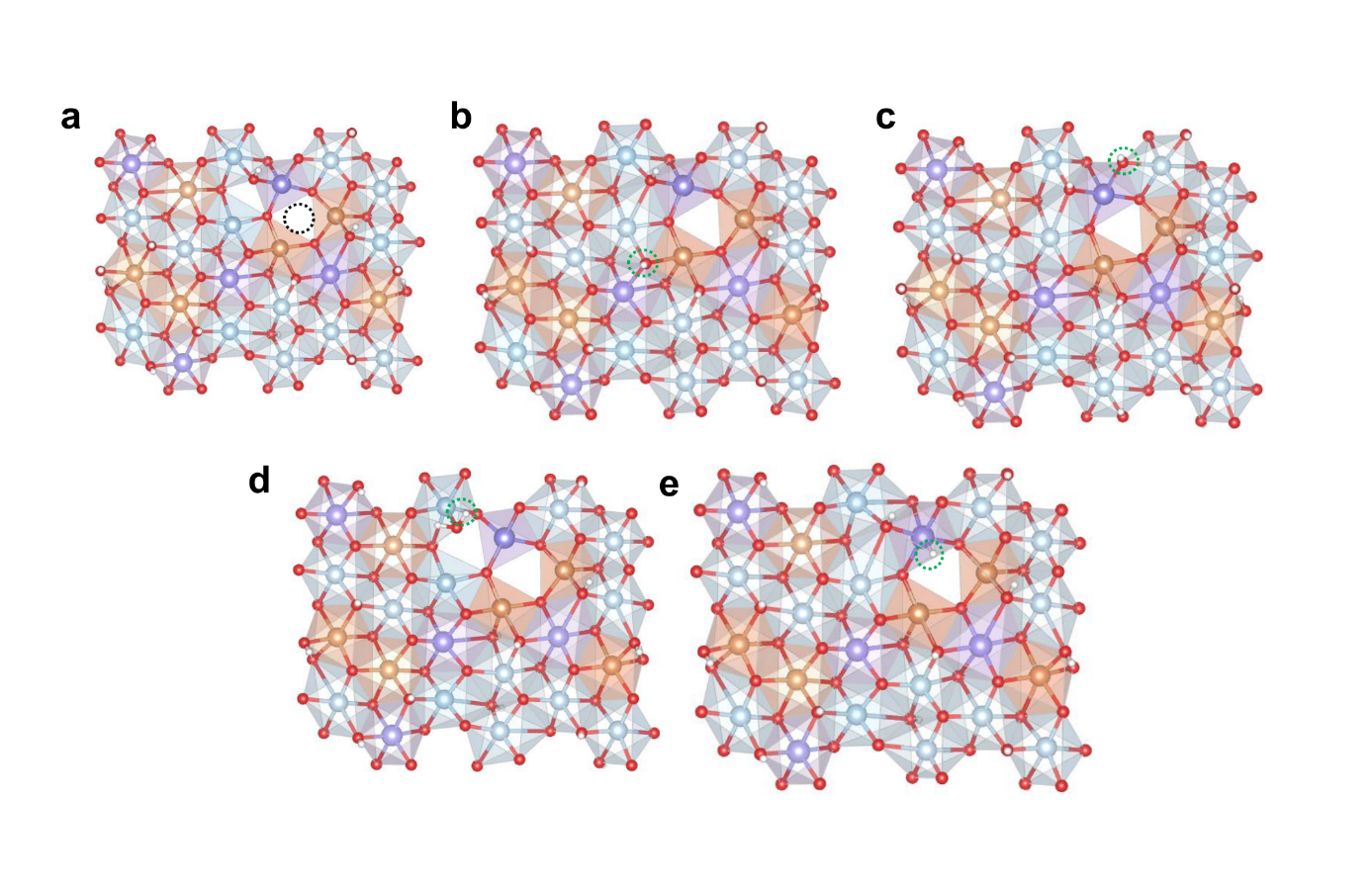


Figure S8. Different positions of hydrogen atom adsorption after dehydrogenation.

A charge density difference analysis was performed to evaluate the charge transfer between the adsorbed states (C_3_H_6_O_3_*, C_3_H_5_O_3_*, and C_3_H_4_O_3_*) and the simulation model (**Figure S5**). A charge transfer of 0.0247 electrons was observed between C_3_H_6_O_3_* and the simulation model, signifying a successful adsorption and catalytic potential. Notably, a stronger charge density difference of 0.6339 electrons was observed for the intermediate C_3_H_5_O_3_*, suggesting enhanced adsorption, which could facilitate catalytic activity. Conversely, the C_3_H_4_O_3_*-adsorbed state demonstrated minimal charge transfer in the model, which is advantageous for the desorption of pyruvic acid, thereby completing the catalytic reaction pathway.


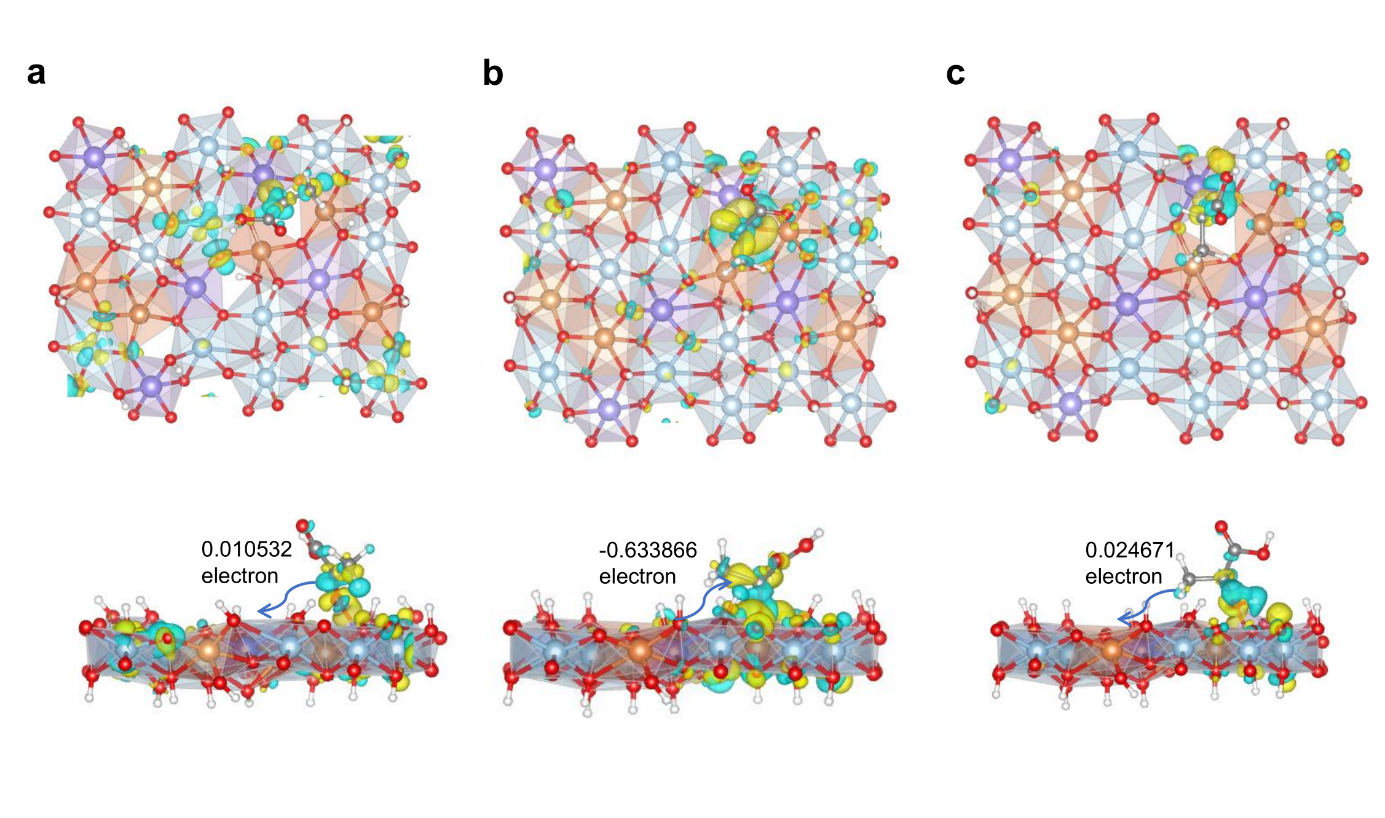


Figure S9. Difference charge density and Bader charge analysis of LDO model.

Comparison of metabolic profiles between normal eye cells (ARPE19) and UM cells (OMM2.3) highlights the higher levels of lactic acid in cancerous cells (**Figure S6a**). Data were analyzed in five replicates. Direct quantification of lactic acid (**Figure S6b**) confirmed increased lactic acid levels in UM cells. Data represent five biological replicates. Comparative analysis of lactic acid concentrations (**Figure S6c**) across a panel of ocular cell lines (normal ARPE19; UM MEL290, MUM2B, OMM2.3, and 92.1), revealing a consistent increase in malignant melanocytes.


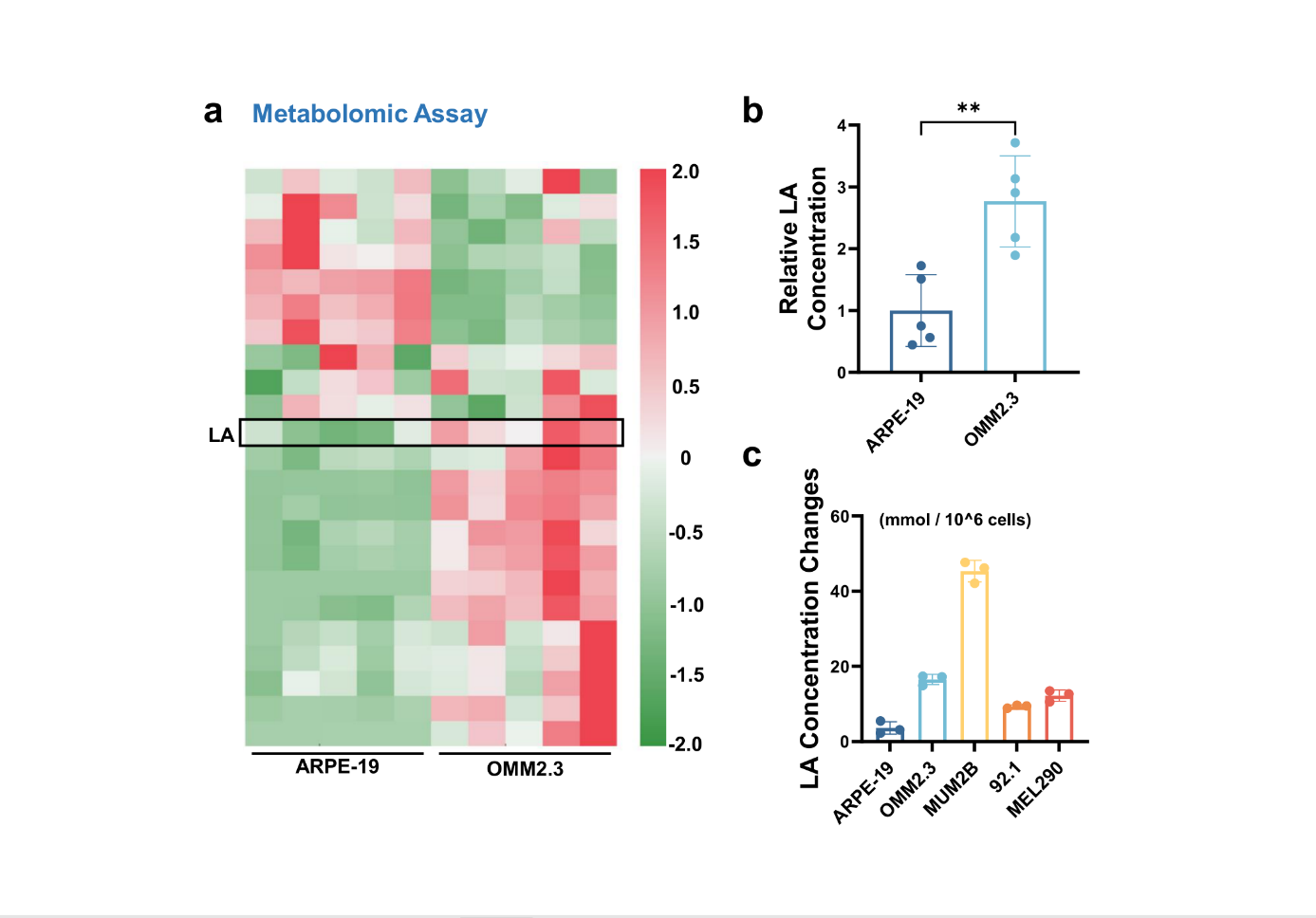


Figure S10. Elevated lactic acid levels in UM cells. (a) Metabolic profiles of normal eye cells (ARPE19) and UM cells (OMM2.3) (b) Direct quantification of lactic acid. (c) Lactic acid concentrations of ARPE19, UM MEL290, MUM2B, OMM2.3, and 92.1. Data were obtained from three replicates. *Abbreviations: LA: lactic acid; RT: radiotherapy*


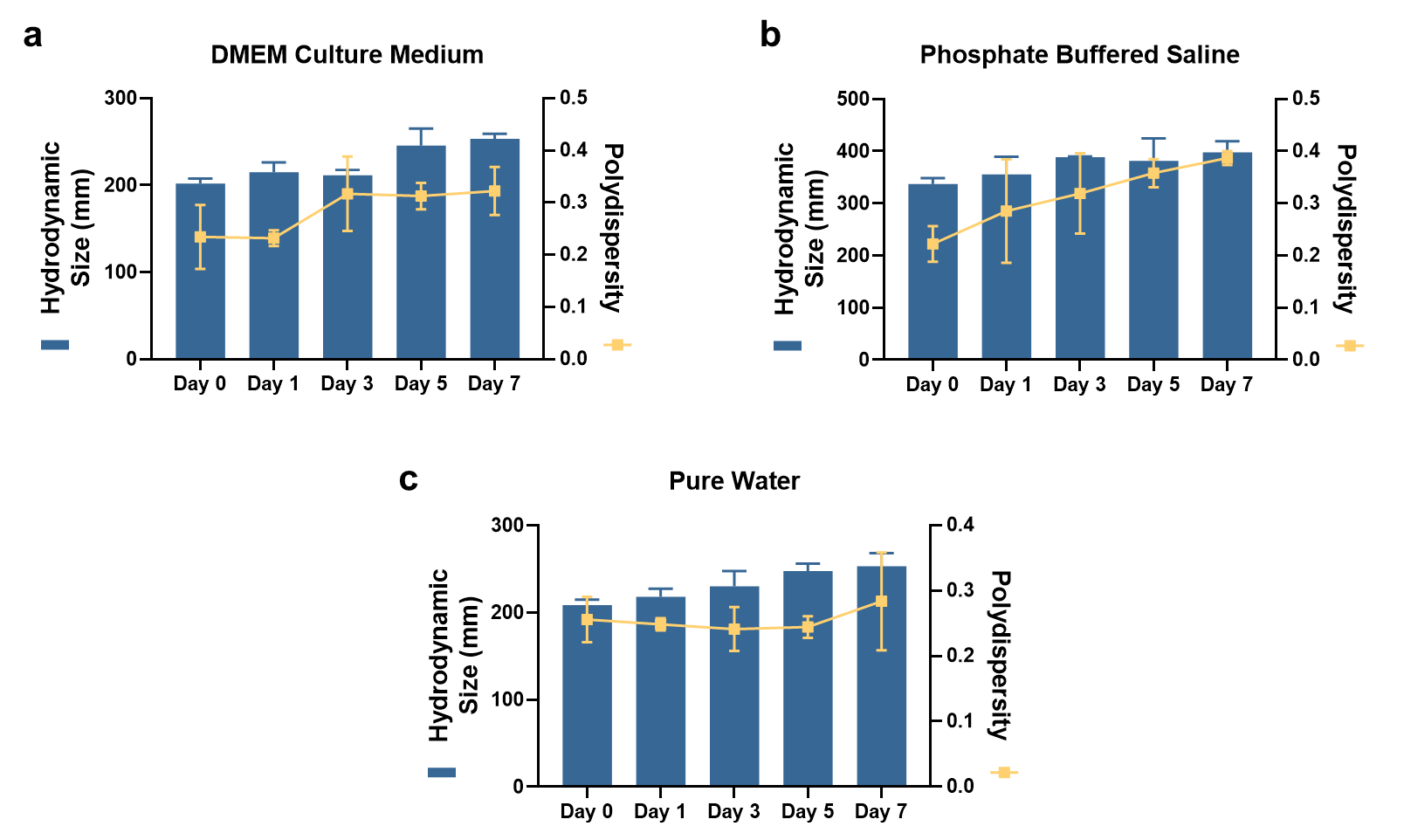


Figure S11. Determination of Hydrodynamic Size and Polydispersity Index by Dynamic Light Scattering. (a) LDO co-incubated with DMEM culture medium. (b) LDO co-incubated with phosphate buffered saline. (c) LDO co-incubated with pure water.

Live/dead staining shows how cells with higher lactic acid are more resistant to radiotherapy. Example images from three replicates are included (**Figure S12a**). Colony formation assays further support the link between elevated lactic acid levels and radiotherapy resistance (**Figure S12b**).


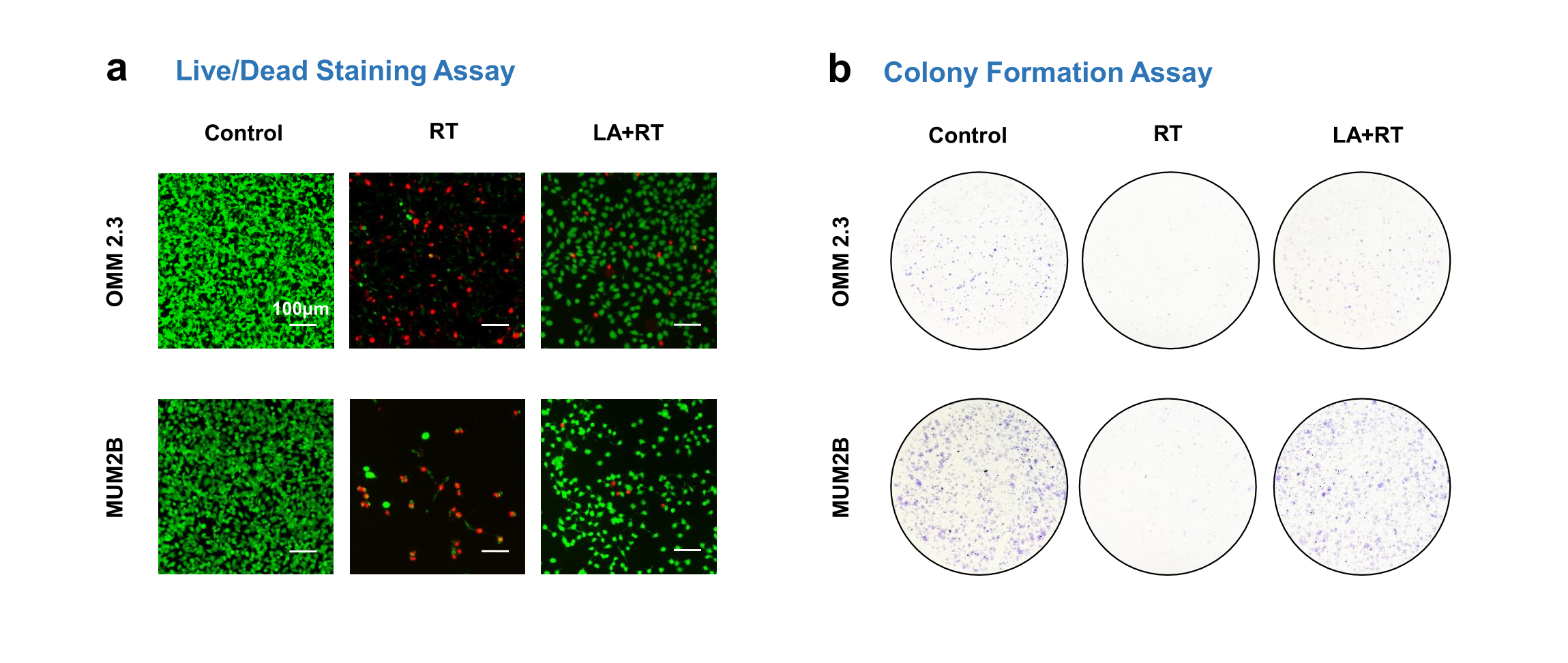


Figure S12. Association of elevated lactic acid levels with radiotherapy resistance in UM cells. (a) Live/dead staining. (b) Colony formation assays. Representative images of three experimental replicates are shown.


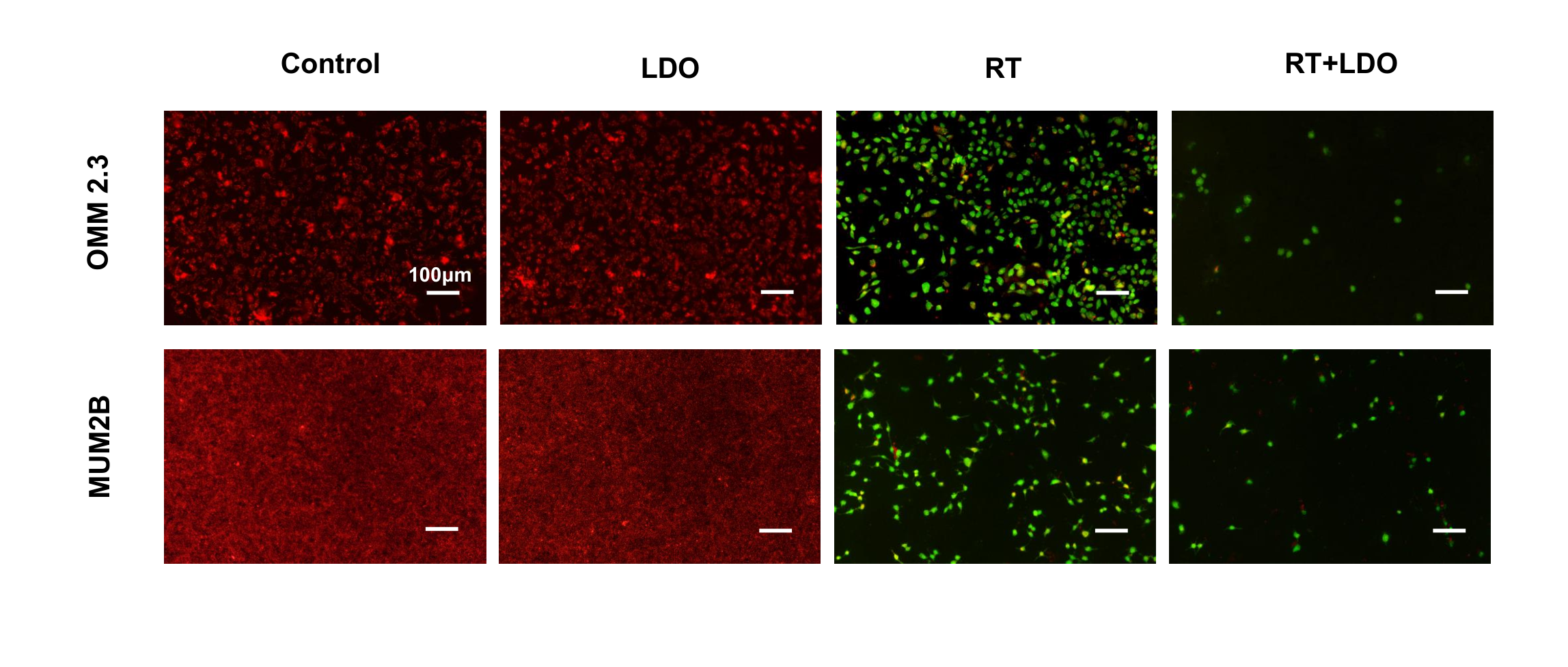


Figure S13. JC-1 staining of OMM2.3 and MUM2B cells. Representative images of three experimental replicates are shown.


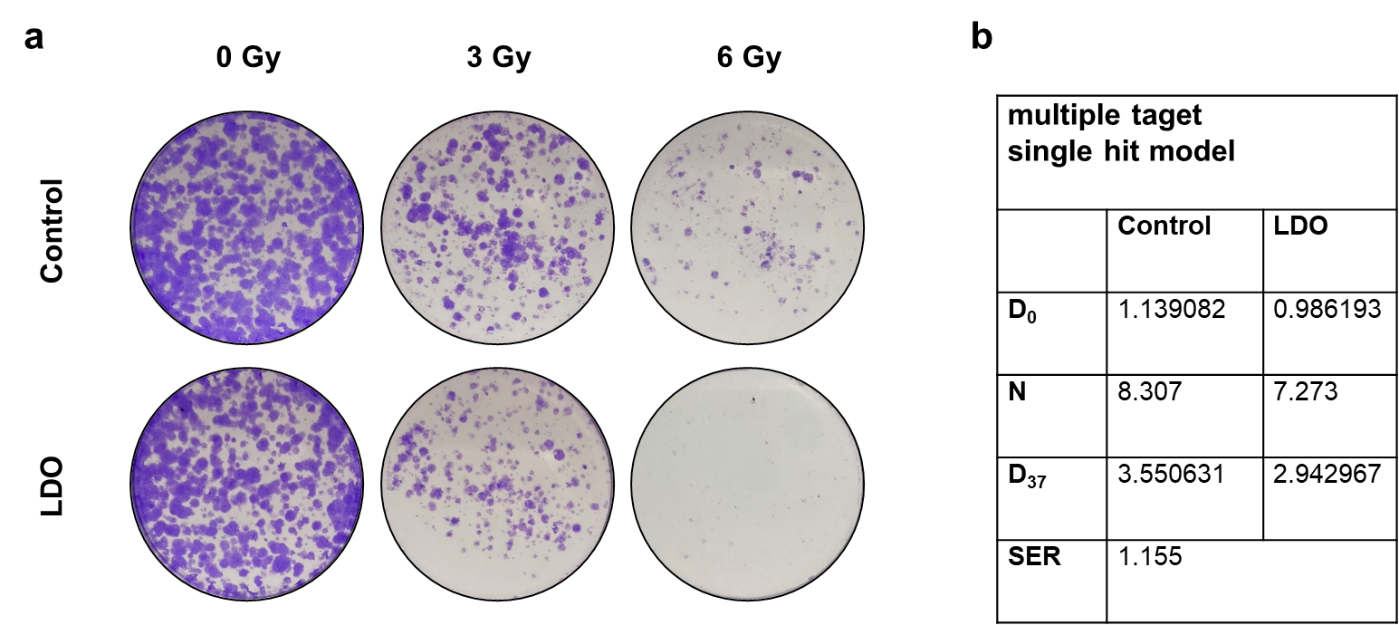


Figure S14. Multi-target single-hit model. (a) Colony formation assays. (b) The radiobiological parameters of the model. Representative images of three experimental replicates are shown.

To further validate its radiosensitizing effects, we extended our study to include human glioblastoma cells (U251) and human lung adenocarcinoma cells (A549), which represent a diverse array of cancers that could benefit from enhanced radiotherapy outcomes. The results showed LDO holds promise for enhancing radiotherapy across a wide variety of cancer types (**Figure S15**).


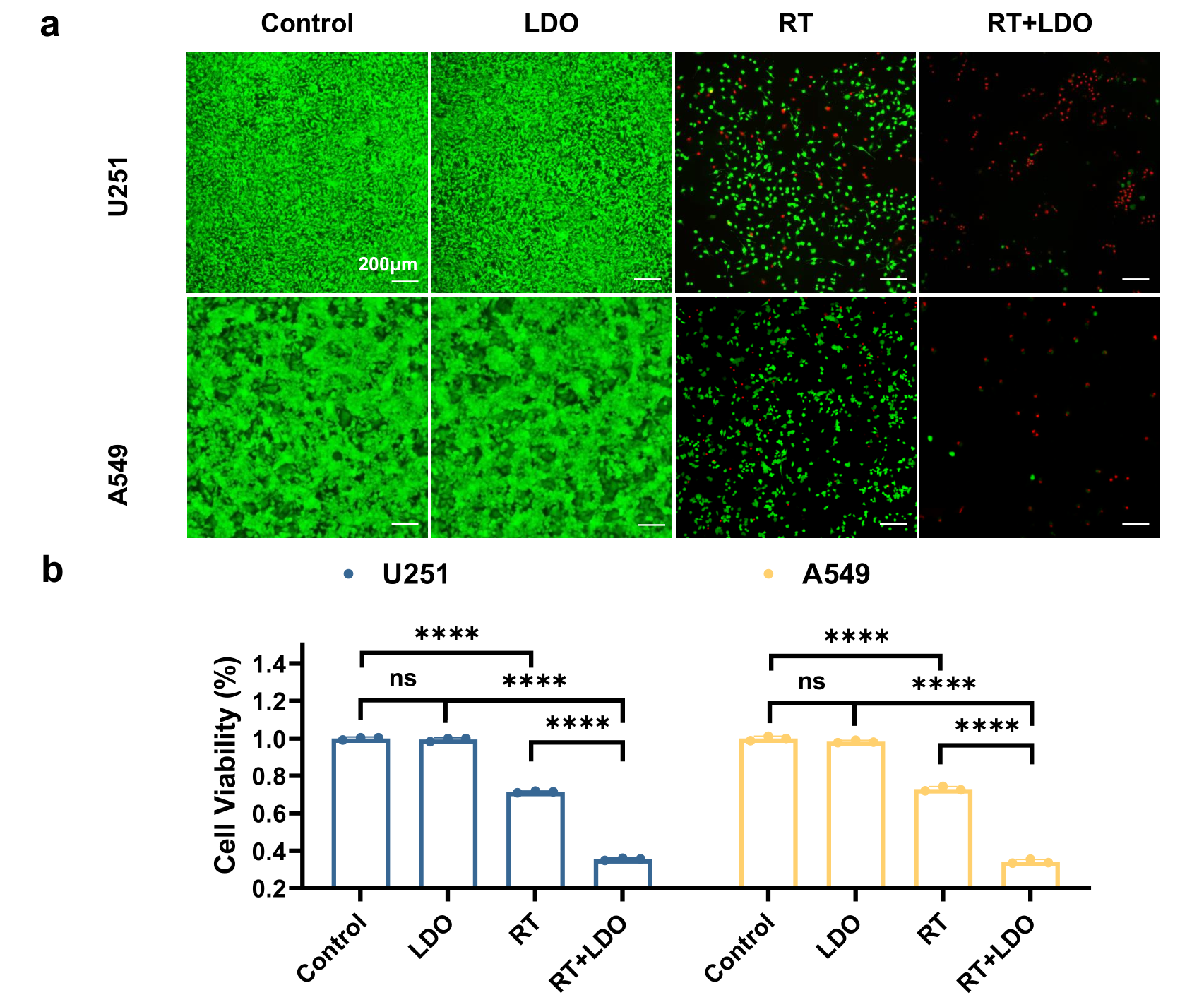


Figure S15. Radiotherapy enhancement by LDO in U251 and A549. (a) CCK8 assay. (b) live/dead cell staining. Representative images of three experimental replicates are shown.


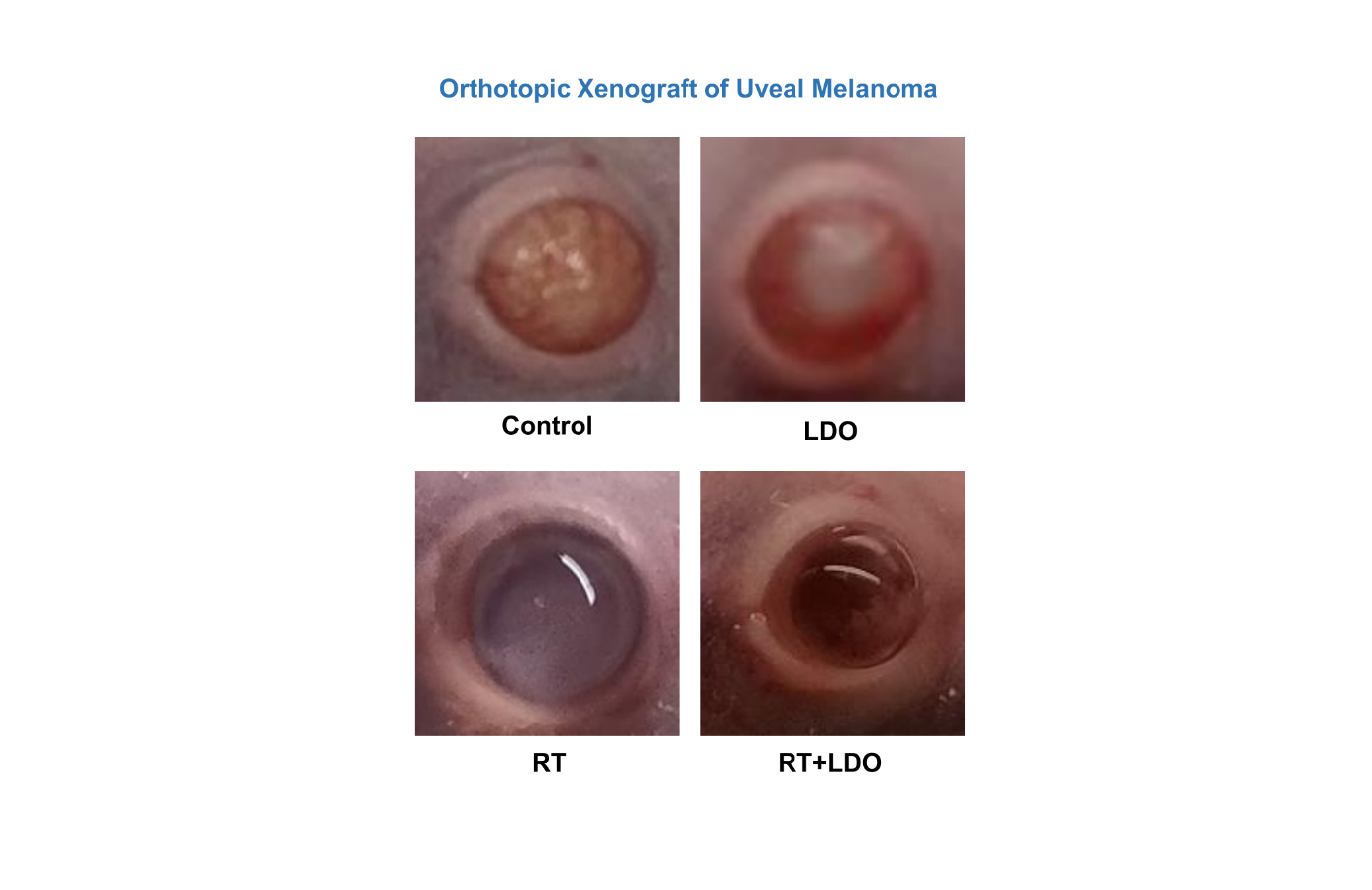


Figure S16. Representative images depicting the macroscopic appearance of the mouse eyes in each treatment group.

A separate subcutaneous tumor model was established to determine metabolic conditions among the treatment groups. Metabolic examination of subcutaneous tumors in nude mice further corroborated the unique capability of LDO to attenuate the lactic acid elevation typically induced by radiotherapy (**Figure S17**), indicating that a critical mechanism underlies the radiosensitizing effects of LDO.


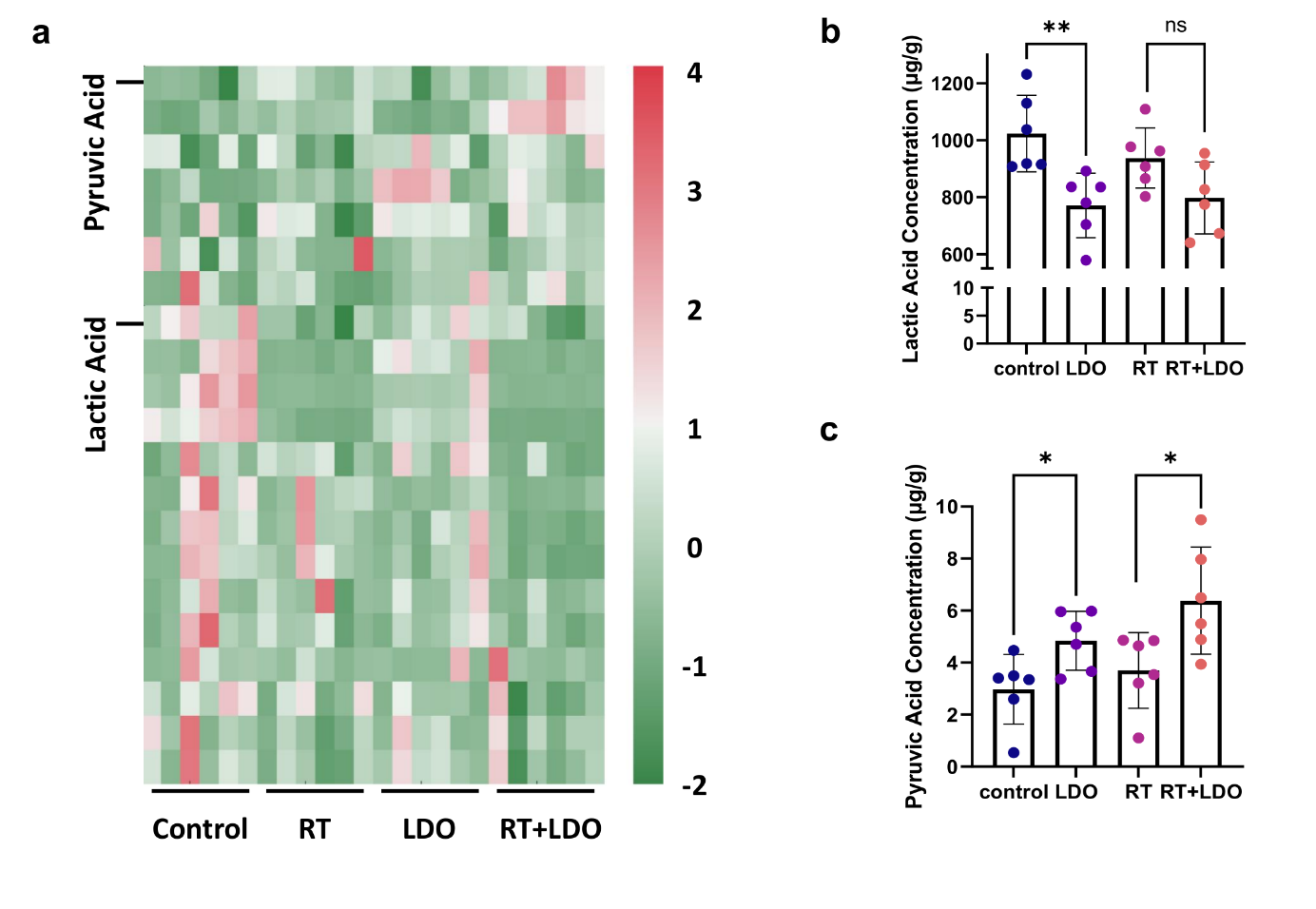


Figure S17. Metabolomics analysis of subcutaneous tumors in nude mice.


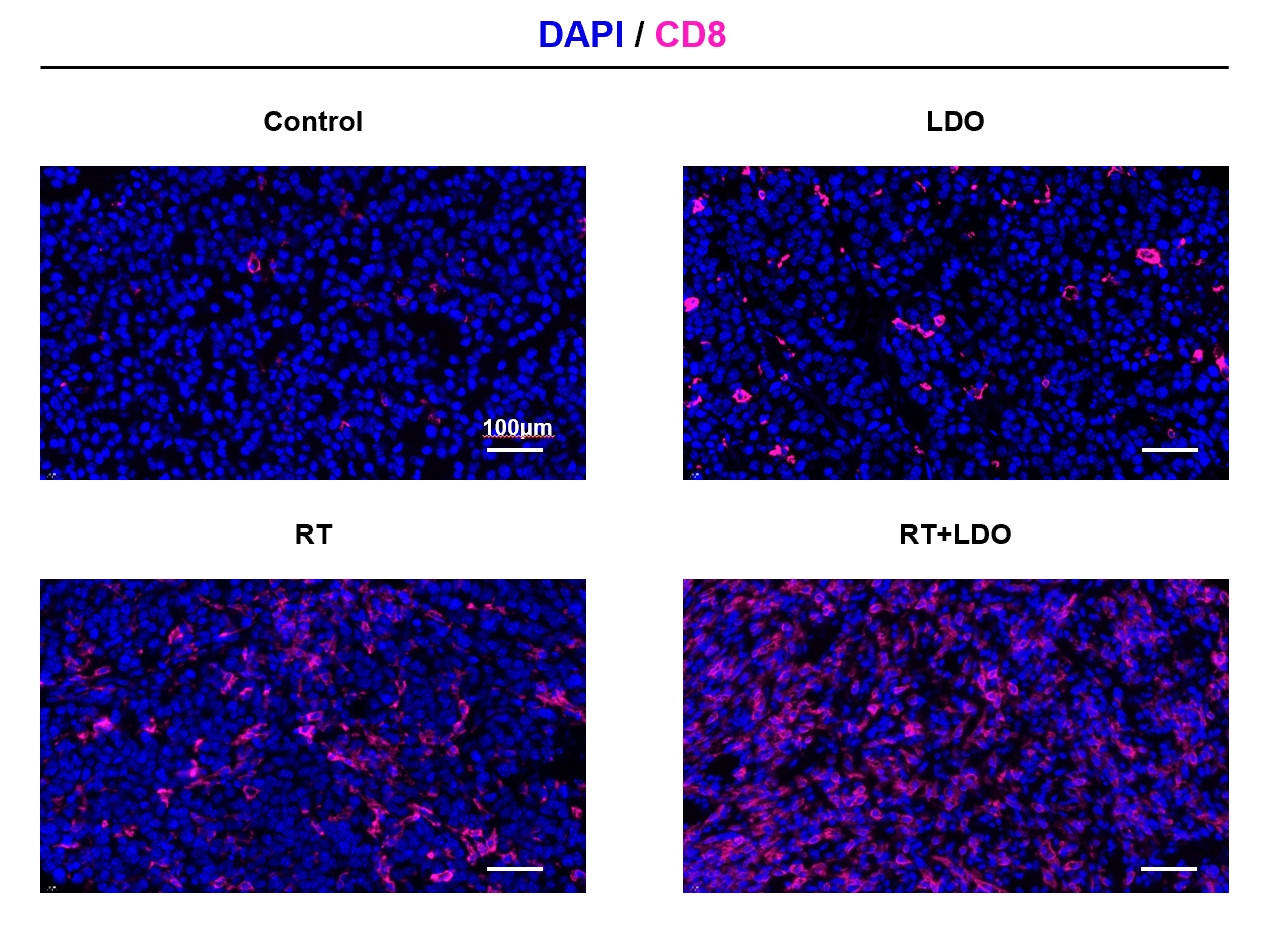


Figure S18. CD8^+^T lymphocyte infiltration
